# Temporal dynamics of stress response in *Halomonas elongata* to NaCl shock: physiological, metabolomic, and transcriptomic insights

**DOI:** 10.1186/s12934-024-02358-5

**Published:** 2024-03-23

**Authors:** Junxiong Yu, Yue Zhang, Hao Liu, Yuxuan Liu, Ali Mohsin, Zebo Liu, Yanning Zheng, Jianmin Xing, Jing Han, Yingping Zhuang, Meijin Guo, Zejian Wang

**Affiliations:** 1 State Key Laboratory of Bioreactor Engineering, East China University of Science and Technology, 130 Meilong Rd, Shanghai, 200237 China; 2 Department of Chemical Engineering for Energy Resources, East China University of Science and Technology, 130 Meilong Rd, Shanghai, 200237 China; 3 State Key Laboratory of Microbial Resources, Institute of Microbiology, Chinese Academy of Sciences, No. 1 Beichen West Road, Chaoyang District, Beijing, 100101 China; 4 Institute of Process Engineering, Chinese Academy of Sciences, Beijing, 100190 PR China

**Keywords:** *Halomonas elongata*, Ectoine, NaCl shock, Stress response, Osmotic stress, Oxidative stress

## Abstract

**Background:**

The halophilic bacterium *Halomonas elongata* is an industrially important strain for ectoine production, with high value and intense research focus. While existing studies primarily delve into the adaptive mechanisms of this bacterium under fixed salt concentrations, there is a notable dearth of attention regarding its response to fluctuating saline environments. Consequently, the stress response of *H. elongata* to salt shock remains inadequately understood.

**Results:**

This study investigated the stress response mechanism of *H. elongata* when exposed to NaCl shock at short- and long-time scales. Results showed that NaCl shock induced two major stresses, namely osmotic stress and oxidative stress. In response to the former, within the cell’s tolerable range (1–8% NaCl shock), *H. elongata* urgently balanced the surging osmotic pressure by uptaking sodium and potassium ions and augmenting intracellular amino acid pools, particularly glutamate and glutamine. However, ectoine content started to increase until 20 min post-shock, rapidly becoming the dominant osmoprotectant, and reaching the maximum productivity (1450 ± 99 mg/L/h). Transcriptomic data also confirmed the delayed response in ectoine biosynthesis, and we speculate that this might be attributed to an intracellular energy crisis caused by NaCl shock. In response to oxidative stress, transcription factor *cys*B was significantly upregulated, positively regulating the sulfur metabolism and cysteine biosynthesis. Furthermore, the upregulation of the crucial peroxidase gene (HELO_RS18165) and the simultaneous enhancement of peroxidase (POD) and catalase (CAT) activities collectively constitute the antioxidant defense in *H. elongata* following shock. When exceeding the tolerance threshold of *H. elongata* (1–13% NaCl shock), the sustained compromised energy status, resulting from the pronounced inhibition of the respiratory chain and ATP synthase, may be a crucial factor leading to the stagnation of both cell growth and ectoine biosynthesis.

**Conclusions:**

This study conducted a comprehensive analysis of *H. elongata*’s stress response to NaCl shock at multiple scales. It extends the understanding of stress response of halophilic bacteria to NaCl shock and provides promising theoretical insights to guide future improvements in optimizing industrial ectoine production.

**Supplementary Information:**

The online version contains supplementary material available at 10.1186/s12934-024-02358-5.

## Background

Halophilic microorganisms are capable of surviving and proliferating in high-salt environments [1, 2]. They possess unique physiological characteristics and adaptive mechanisms to withstand high salt conditions, including “salt-in” and “compatible-solute” [3–5]. A plethora of studies have delved into the salt adaptation mechanisms of halophiles, albeit primarily centered around varying fixed salt concentrations [6–8]. However, these studies may inadequately reflect the true growth conditions, as microorganisms in their natural growth processes undergo increases in osmotic pressure due to prolonged periods of drought or sudden flooding, leading to a sharp decrease in osmotic pressure [9, 10]. On the other hand, the elevated excretion of metabolites and substrate feed will lead to the elevation of osmolarity in biotechnological applications [11].

*Halomonas elongata* is a halophilic γ-proteobacterium [12] that is capable of surviving and proliferating in high-salt environments [13]. *H. elongata* primarily accumulates ectoine, an aspartate derivative (1,4,5,6-tetra-2-methyl-4-pyrimidinecarboxylic acid), as its main compatible solute to sustain osmotic balance [12]. Ectoine is a commercially important compatible solute widely used in medicine and cosmetics [14]. *H. elongata* stands out as a crucial industrial producer of ectoine [15]. Some previous studies have reported the complete genome sequence and salinity adaptation mechanism of *H. elongata* [12, 13, 16, 17]. The glucose degradation most likely proceeds solely via the Entner–Doudoroff (ED) pathway in *H. elongata*, while the Embden-Meyerhof-Parnas (EMP) pathway may not be active [18]. Moreover, the genes encoding chemotaxis and flagellar assembly are strongly upregulated under salt stress [13]. Our previous study also demonstrated the flexible adaptation of *Halomonas elongata* DSM 2581^T^ to varying salt concentrations [19], with the highest biomass and ectoine accumulation achieved at 8% NaCl, accompanied by enhanced ectoine biosynthesis and ED pathway compared to that under 1% NaCl condition [18]. Furthermore, potassium is also found to play essential roles in osmoregulation in *H*. *elongata* [20]. An elevation in cytoplasmic K^+^ and glutamate was observed in *H*. *elongata* when subjected to an osmotic upshock (3–6% NaCl) [20]. However, beyond this, few studies have paid attention to the response of *H. elongata* to rapid changes in environmental osmolarity.

The cellular stress response to salt shock is usually a multi-phased process [9], microorganisms have to regulate water movement across their cytoplasmic membrane by increasing cytoplasmic osmotic potential. Many microorganisms swiftly uptake K^+^ ions as an emergency reaction after salt shock [21]. Subsequently, this ion is substituted with compatible solutes, a category of organic osmolytes that closely align with cellular physiology [22]. Moreover, the biosynthesis and absorption of compatible solutes such as betaine and proline are pivotal for *Bacillus subtilis* to defend against the challenges posed by high salinity [23]. Another study revealed that the differentially expressed genes associated with “defense mechanisms” and “nucleotide transport and metabolism” were identified as upregulated upon analyzing the transcriptional response of *Mesorhizobium loti* to salt shock [24]. Additionally, the oxidative stress induced by salt shock is another severe challenge [25], elevating intracellular Reactive Oxygen Species (ROS) levels and causing cell structural damage. Therefore, the overall adjustment process to salt shock is a rather complex process. There are still many open questions about how *H*. *elongata* addresses the challenges posed by NaCl shock and the mechanisms it employs to maintain cellular homeostasis under such conditions.

In the present study, the physiological and metabolic responses of *H. elongata* to NaCl shock at short- and long-time scales were investigated in detail. Moreover, transcriptome and metabolite analysis were performed to further explore the stress-responsive mechanisms of *H. elongata*. Finally, the effective strategies to protect cells from shock treatment were also examined. In summary, this study provided a multi-scale and multi-parameter analysis of the stress response of *H. elongata* to NaCl shock, proposed protective strategies, and contributed to a deeper understanding of the physiological and molecular processes underlying the NaCl shock response in halophilic microorganisms.

## Results

### Physiological response of *H. elongata* to NaCl shock

**Fig. 1:**
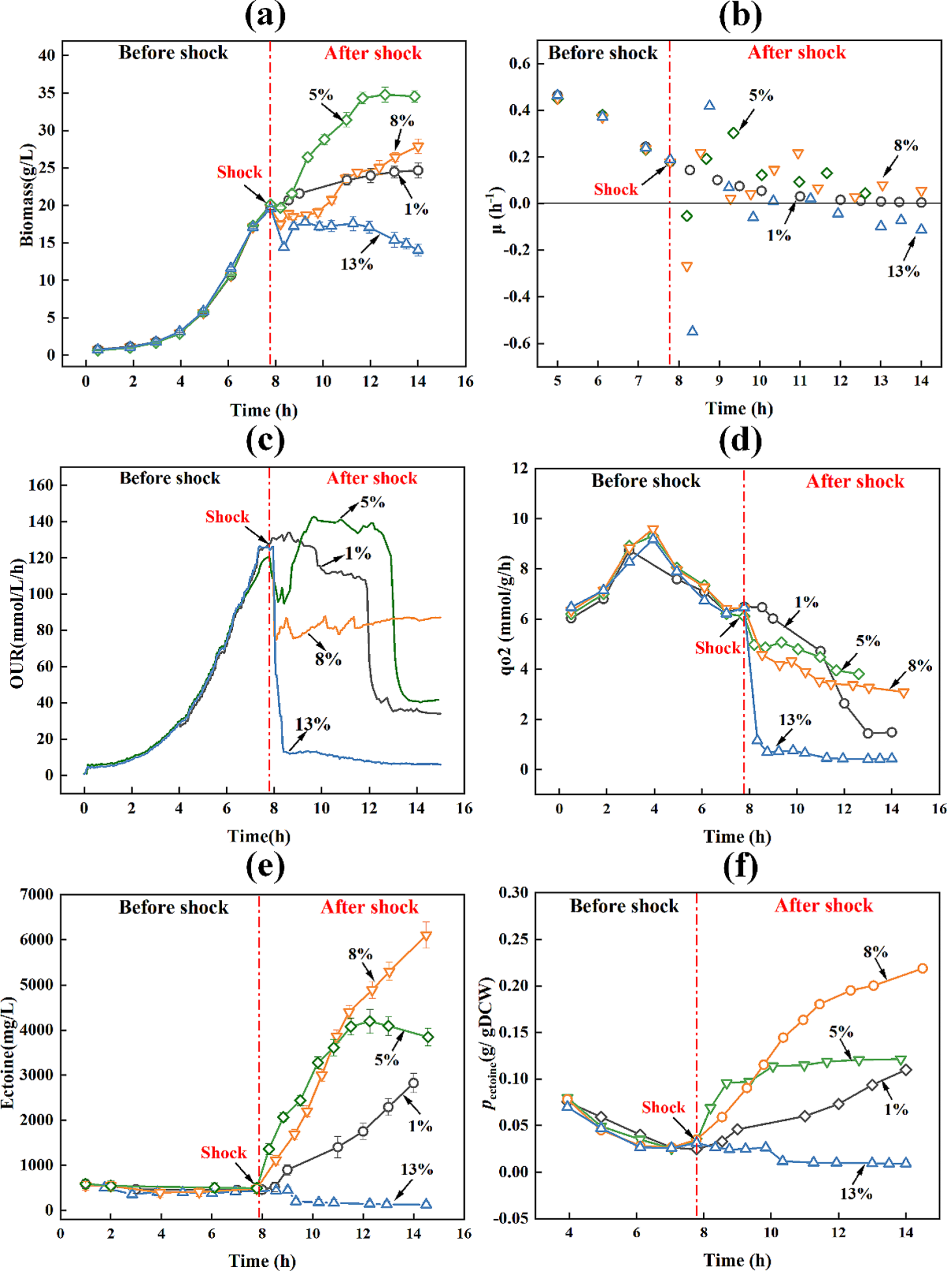
Changes in physiological parameters of *H. elongata* after NaCl shock. Biomass**(a)**, the specific growth rate (*µ*) **(b)**, oxygen uptake rate **(c)**, the specific oxygen uptake rate (*q*_*O2*_) **(d)**, ectoine production**(e)**, ectoine produced per biomass (*p*_ectoine_) **(f)**. The numbers inside the figure indicate the NaCl concentration in fermentation broth after shock

As shown in Fig. 1, *H.elongata* exhibited differential responses in physiological metabolism to osmotic shock induced by various NaCl concentrations. First, the change in biomass indicated that the NaCl shock had significant effects on cell growth (Fig. 1a), as there was a consistent decrease in biomass within 1 h after NaCl shock across all treatment groups. The 5% NaCl shock treatment induced one hour cell growth arrest, followed by a faster growth rate than the control group. Moreover, the biomass reached 31.41 ± 0.96 g/L at the fourth hour after shock, which was 34% higher than that of the control group (Table 1). Similarly, the 5% NaCl shock induced a decrease of 20.8% in the oxygen uptake rate (OUR) initially, followed by a rapid increase after 1 h (Fig. 1c). When the NaCl concentration was raised to 8%, *H. elongata* took more time to adapt to the hypertonic environment, and the biomass started to increase after 2 h following the shock. Additionally, following the 8% NaCl shock, OUR decreased rapidly from 127.83 ± 5.43 to 77.91 ± 9.54 mmol/L/h, with no significant subsequent increase (Fig. 1c). Strong inhibitory effects on cell growth and respiration were observed under 13% NaCl shock treatment, with the biomass and OUR decreasing by 12% and 90%, respectively, without recovery. In addition, the specific oxygen uptake rate (*q*_*O2*_) values gradually decreased with increasing NaCl shock concentrations (Fig. 1d). Besides, the glucose consumption rates were drastically affected by NaCl shock treatments. At 4 h after shock treatment, the residual glucose contents in the 5%, 8% and 13% NaCl were 1.82 ± 0.31, 8.71 ± 0.61 and 19.12 ± 0.68 g/L, respectively, whereas in the control group, this value was only 0.66 ± 0.11 g/L(Table 1). Moreover, the sudden decrease in OUR at around 5 h following 5% NaCl shock was attributed to glucose depletion. Likewise, glucose depletion accounted for the decline in OUR and *q*_*O2*_ at 12 h in the control group (Fig. 1c).

**Table 1:** Physiological metabolic parameters of *H. elongata* at 4 h after NaCl shock

Parameters	Before shock	Control	5%^b^	8%^b^	13%^b^
OUR (mmol/L/h)	127.83 ± 5.43	107.83 ± 8.78	137.69 ± 9.54	77.91 ± 9.54	9.89 ± 1.21
*q*_o2_ (mmol/g/h)	6.53 ± 0.18	4.61 ± 0.13	4.38 ± 0.12	3.29 ± 0.11	0.57 ± 0.08
CER (mmol/L/h)	130.15 ± 6.23	110.34 ± 5.65	145.82 ± 7.64	82.42 ± 6.87	11.21 ± 1.62
*q*_co2_ (mmol/g/h)	6.65 ± 0.21	4.72 ± 0.14	4.64 ± 0.15	3.48 ± 0.15	0.64 ± 0.06
Biomass (g/L)	19.58 ± 0.23	23.38 ± 0.57	31.41 ± 0.96	23.65 ± 0.78	17.38 ± 0.66
*µ* (h^− 1^)	0.18 ± 0.02	0.06 ± 0.01	0.13 ± 0.01	0.05 ± 0.01	0.02 ± 0.01
P (g/L)	0.54 ± 0.09	1.75 ± 0.23	4.08 ± 0.28	4.58 ± 0.19	0.31 ± 0.06
*q*_p_ (mg ectoine/ g DCW/h)	1.35 ± 0.01	12.13 ± 0.15	12.41 ± 0.32	66.54 ± 1.86	0.00 ± 0.00
Glucose (g/L)	21.65 ± 0.65	0.66 ± 0.11	1.82 ± 0.31	8.71 ± 0.61	19.12 ± 0.68
Productivity^a^ (mg/L/h)	30 ± 11	320 ± 61	1230 ± 112	1450 ± 99	-40 ± 10

Productivity^a^ refers to the maximum productivity during 4 h after NaCl shock

^b^ refers to the NaCl concentration (%) in the fermentation broth after osmotic shock

CER (carbon dioxide expiration rate), OUR (oxygen uptake rate)

Further, results showed that 5% and 8% NaCl shock treatments induced a rapid biosynthesis rate and accumulation of intracellular ectoine (Fig. 1e), which reached 4.08 ± 0.28 and 4.58 ± 0.19 g/L at 4 h after shock, respectively (Table 1). However, hardly any ectoine accumulated in 13% NaCl shock treatment. Over the 2 h after shock treatment, more ectoine was produced under 5% NaCl shock compared to that of 8% NaCl shock treatment. However, a higher amount of ectoine produced per biomass (*p*_ectoine_) was observed in 8% NaCl shock after 2 h of the shock (Fig. 1f). Additionally, the maximum ectoine productivity in 5% and 8% NaCl shock conditions reached 1230 ± 112 and 1450 ± 99 mg/L/h (Table 1), respectively. Importantly, the specific ectoine production rate (*q*_*p*_) in 8% NaCl shock reached 66.54 mg ectoine/g DCW/h, which was 5.5-fold compared to that of control.

### The differences in carbon source distribution after NaCl shock

Here, the carbon in consumed glucose within 4 h after shock were mainly converted into three parts: biomass, generated CO_2_, and byproduct. Ectoine is an intracellular product in *H. elongata*, this part of carbon is included in biomass. As shown in Table S1, △C (The difference between total carbon in consumed glucose and carbon in generated biomass and CO_2_) was 0.37, -0.77, and 0.00 mol in the control, 5%, and 8% NaCl groups, respectively. It should be mentioned that the medium contained a considerable organic nitrogen source, leading to the results where the generated carbon was greater than the carbon in consumed glucose. Furthermore, without considering byproduct, the proportion of carbon in the generated CO_2_ significantly decreased in both the 5% and 8% NaCl shock groups compared to the control group (decreasing from 80.33 to 60.19% and 70.70%, respectively), while concurrently, the proportion of carbon in the biomass increased from 19.67% to 39.81% and 29.3%, respectively (Additional file 1: Fig. S1).

### Changes in cell morphology after NaCl shock

As shown in Figure S2, the cells without NaCl shock treatment displayed intact rod-like morphology. At 10 min after 8% and 13% NaCl shock, cells with irregular morphology were observed, particularly in the 13% NaCl shock group, where a larger number of damaged cells and cell debris appeared (Additional file 1: Fig. S2). Additionally, arrowheads indicated the presence of ruffles on the surface of the cells after 13% NaCl shock (Additional file 1: Fig. S2).

### Changes in intracellular sodium and potassium ion content after NaCl shock

**Fig. 2:**
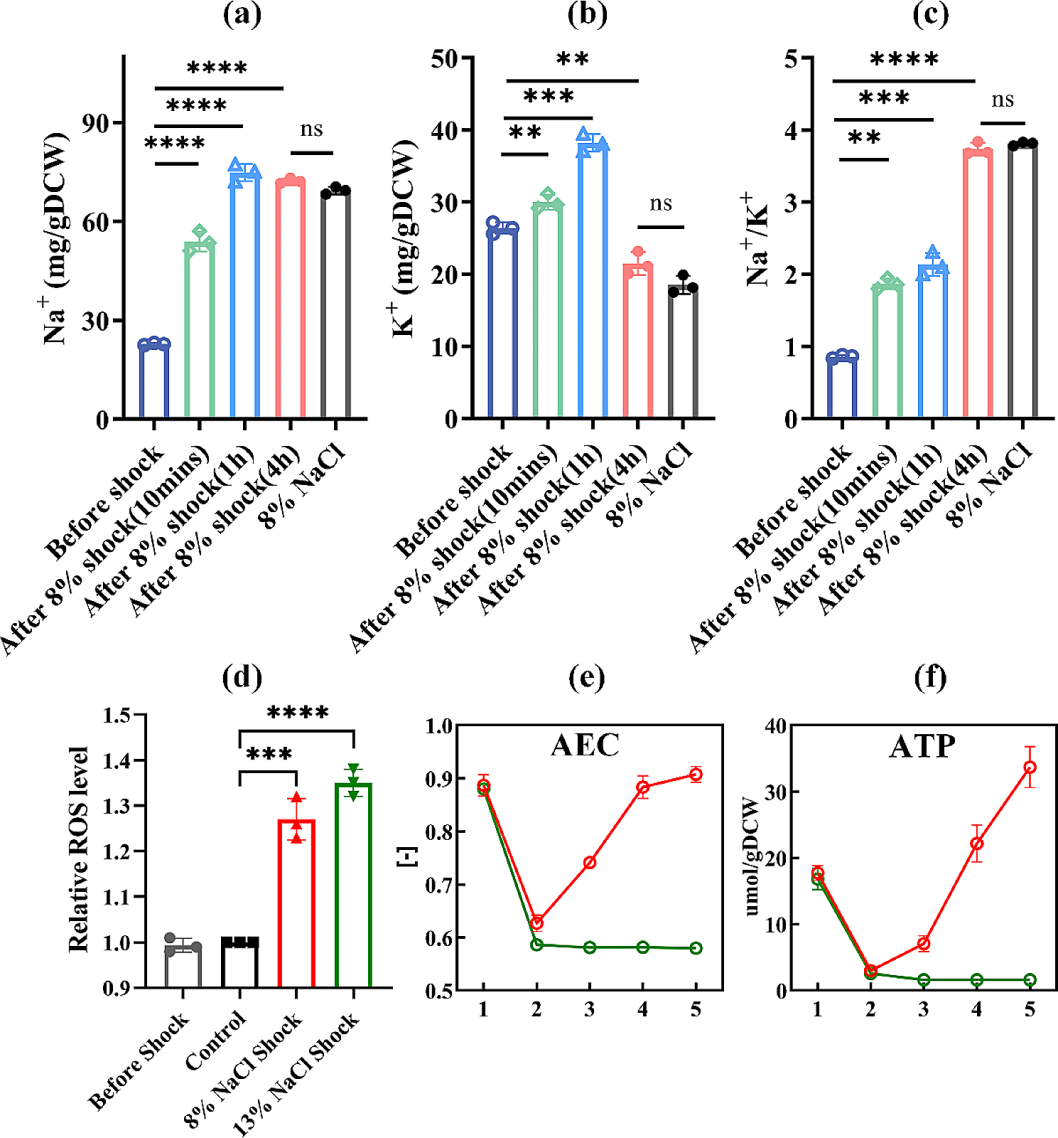
Changes in intracellular sodium **(a)**, potassium **(b)** ion and Na^+^/K^+^ ratio **(c)** levels after 8% NaCl shock. Where the “8% NaCl” represents the condition in fixed 8% NaCl. And the ROS levels **(d)**, AEC ratio **(e)**, and ATP contents **(f)** were also determined. The numbers (1, 2, 3, 4, and 5) on the x-axis in panels (e) and (f) represent different sampling time, which are 5 min before shock, 5, 10, 20, and 30 min after shock, respectively. Red lines indicate 8% NaCl shock group, green lines indicate 13% NaCl shock group. ** represents *p* < 0.01, *** represents *p* < 0.0005, **** represents *p* < 0.0001

Intracellular Na^+^ and K^+^ contents were detected at 10 min, 1 h, and 4 h after 8% NaCl shock. Simultaneously, measurements were also taken for the control group and the log-phase cells under 8% NaCl stress. As shown in Fig. 2, compared to the levels before the shock, Na^+^ and K^+^ contents increased by 136.4% and 13.7% at 10 min after 8% NaCl shock, respectively. Specifically, Na^+^ content increased from 22.81 ± 0.24 to 53.89 ± 2.47 mg/gDCW, and K^+^ content increased from 26.41 ± 0.65 to 30.01 ± 0.86 mg/gDCW (Fig. 2a, b). And their contents peaked at 1 h after shock, reaching 74.84 ± 2.14 (Na^+^) and 38.23 ± 0.98 (K^+^) mg/gDCW, respectively. It was noteworthy that one hour after the shock, the K^+^ content was 2.1 times higher than that in the 8% NaCl stress condition. As time progressed, the change in Na^+^ content was not significant, while the K^+^ content dropped to 21.47 ± 0.72 mg/gDCW at 4 h after shock, with no significant difference with that in the 8% NaCl stress condition. Additionally, the Na^+^/ K^+^ ratio increased from 0.86 (before shock) to 3.74 (4 h after shock) (Fig. 2c).

### Changes in intracellular metabolite pools of *H. elongata* within short timescales after NaCl shock

**Fig. 3:**
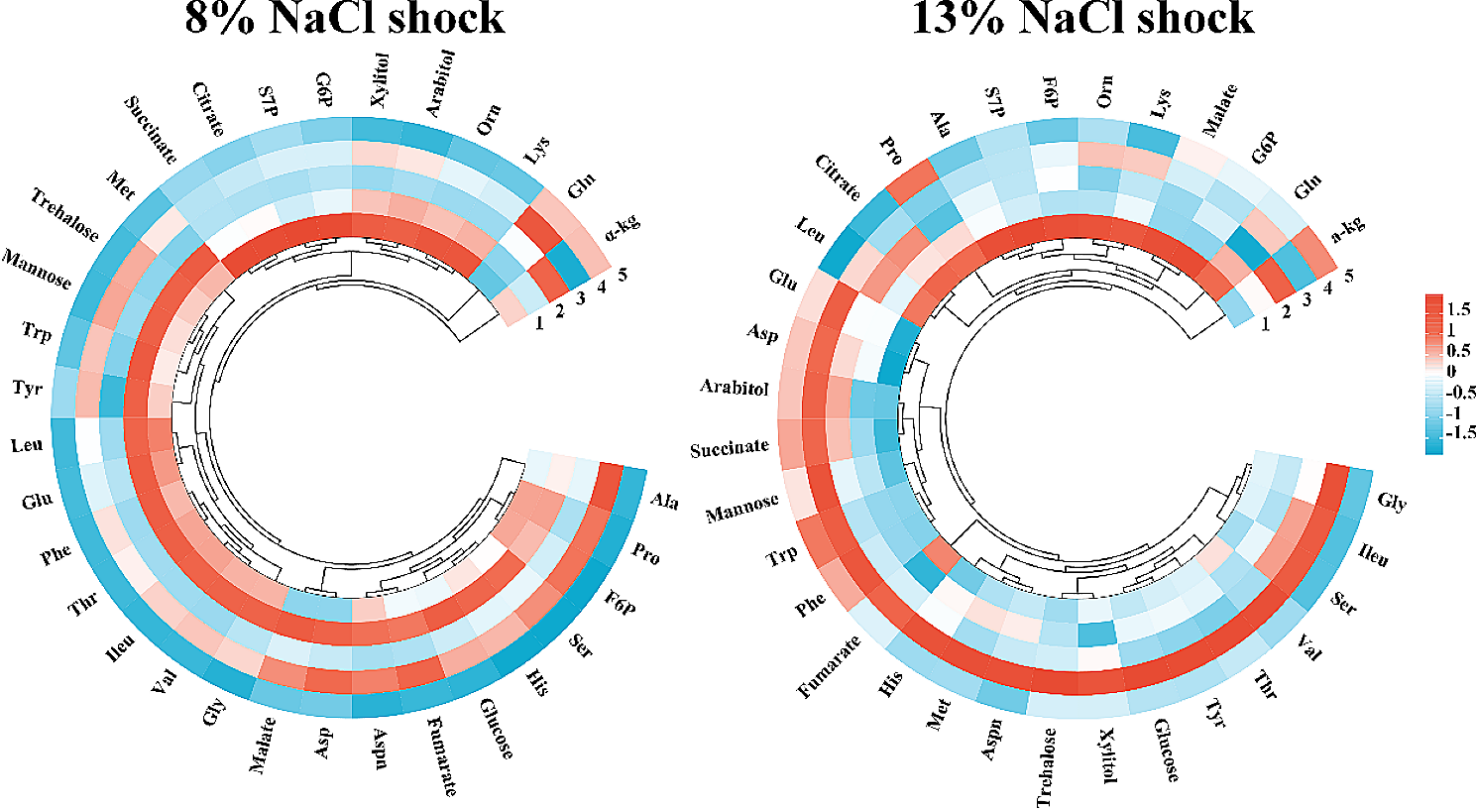
Heatmap for metabolite changes in *H. elongata* after 8% and 13% NaCl shock, and color-scaled with the contents of metabolites. The number (1, 2, 3, 4, and 5) in the heatmap indicates different sampling time, which are 5 min before shock, 5, 10, 20, and 30 min after shock, respectively

In the preceding section, the physiological responses of *H. elongata* were demonstrated at long-time scales following NaCl shock. Furthermore, particular attention was directed towards the responses occurring at short timescales after the shock. Results showed that ectoine accumulation showed no statistically significant change within 20 min after 8% NaCl shock (Additional file 1: Fig. S3a). To better understand how *H. elongata* cells cope with external upshift osmolality during this period time, the intracellular metabolite pools of *H. elongata* were measured under 8% and 13% NaCl shock treatments within 30 min after shock. The heatmaps displayed the changes of 32 metabolites (including amino acids, organic acids, sugar alcohols, and sugar phosphates) during short timescales (5 min before shock, 5, 10, 20, and 30 min after shock) (Fig. 3). Obviously, the intracellular metabolite pools exhibited significant differences in the change trends under different NaCl shock treatments.

Fifteen amino acid pools in the 8% NaCl shock treatment increased at 5 min after the shock. Notably, aspartate, glutamate, glutamine, and threonine concentrations showed a dramatic increase inside the cells, while the phenylalanine, lysine, and ornithine concentrations decreased significantly (Additional file 1: Fig. S4). Aspartate, an important precursor for ectoine biosynthesis, exhibited a 150% increase in intracellular content, rising from 0.08 to 0.2 mg/g DCW at 5 min after the shock (Additional file 1: Fig. S4). Additionally, glutamine and glutamate contents also showed marked increases of 40% and 90%, respectively, at 5 min after 8% NaCl shock compared to before shock. The change in intracellular pools of glutamine was of utmost concern, increasing from 5.7 mg/g DCW before shock to 57.3 mg/g DCW after 20 min under 8% NaCl shock, with an elevation of 905%. However, after 20 min, the majority of amino acids showed a significant decrease in their content.

When the NaCl shock concentration was increased to 13%, aspartate, asparagine, histidine, methionine, and glutamate exhibited increased pools at 5 min after the shock (Additional file 1: Fig. S4). Additionally, there was no significant change in glutamine content after the shock, which differed from the response observed in the 8% NaCl shock group. Furthermore, most amino acids content exhibited an increase at 20 min after shock. As depicted in Figure S4, a notable elevation in the intracellular pools of storage sugars (arabinitol, mannitol, and trehalose) was observed at 20 min after 13% NaCl shock treatment. Specifically, the contents of mannitol and trehalose increased by 40% and 43%, respectively. However, this phenomenon was not observed in the 8% NaCl shock treatment. In addition, some sugar phosphates (glucose-6-phosphate, fructose-6-phosphate, and sedoheptulose-7-phosphate) showed a declining trend after shock compared to their levels before shock, which can be attributed to an increase in the metabolic pool of amino acids.

### Changes in energy and ROS levels of *H. elongata* after NaCl shock

Under the NaCl shock treatment, changes in physiological parameters indicated that cell growth and respiration were suppressed within 30 min (Fig. 1), resulting in a rapid decline in the adenylate energy charge (AEC) ratio, which is related to cellular energy status (Fig. 2e). The intracellular ATP content decreased from 17.7 ± 1.1 and 16.8 ± 1.6 µmol/ gDCW to 3.0 ± 0.5 and 2.6 ± 0.3 µmol/ gDCW at 5 min after shock treatment in 8% and 13% NaCl shock groups, respectively (Additional file 1: Table S2). Subsequently, the ATP content in 8% NaCl shock group increased rapidly and reached a higher level (22.2 ± 2.8 µmol/ gDCW) at 20 min after the shock compared to before the shock treatment(Fig. 2f). As a result, the cells recovered to the normal energy status (AEC = 0.88). However, in the 13% NaCl shock group, both ATP content and AEC ratio did not show recovery to pre-shock levels for at least 30 min (AEC<0.6). Additionally, the intracellular ROS levels were measured at 10 min after shock. The results revealed a significant increase in ROS levels within both the 8% and 13% NaCl shock groups (Fig. 2d). Moreover, concurrently, there was a significant increase in malondialdehyde (MDA) content (*p*<0.01) (Additional file 1: Fig. S3d).

### Transcriptome profiling of *H. elongata* after NaCl shock

The metabolite analysis revealed significant difference in *H. elongata* cells under 8% and 13% NaCl shocks within a short time (30 min). To further understand the metabolic regulation, the study investigated the differentially expressed genes (DEGs) in *H. elongata* under these two shock conditions. mRNAs from the five samples (5 min before shock, 5 and 10 min after shock for both shock groups, respectively) were sequenced. Fifteen cDNA libraries were constructed and sequenced on Illumina HiSeq X Ten (2 × 150 bp) (Additional file 1: Table S3).

**Fig. 4:**
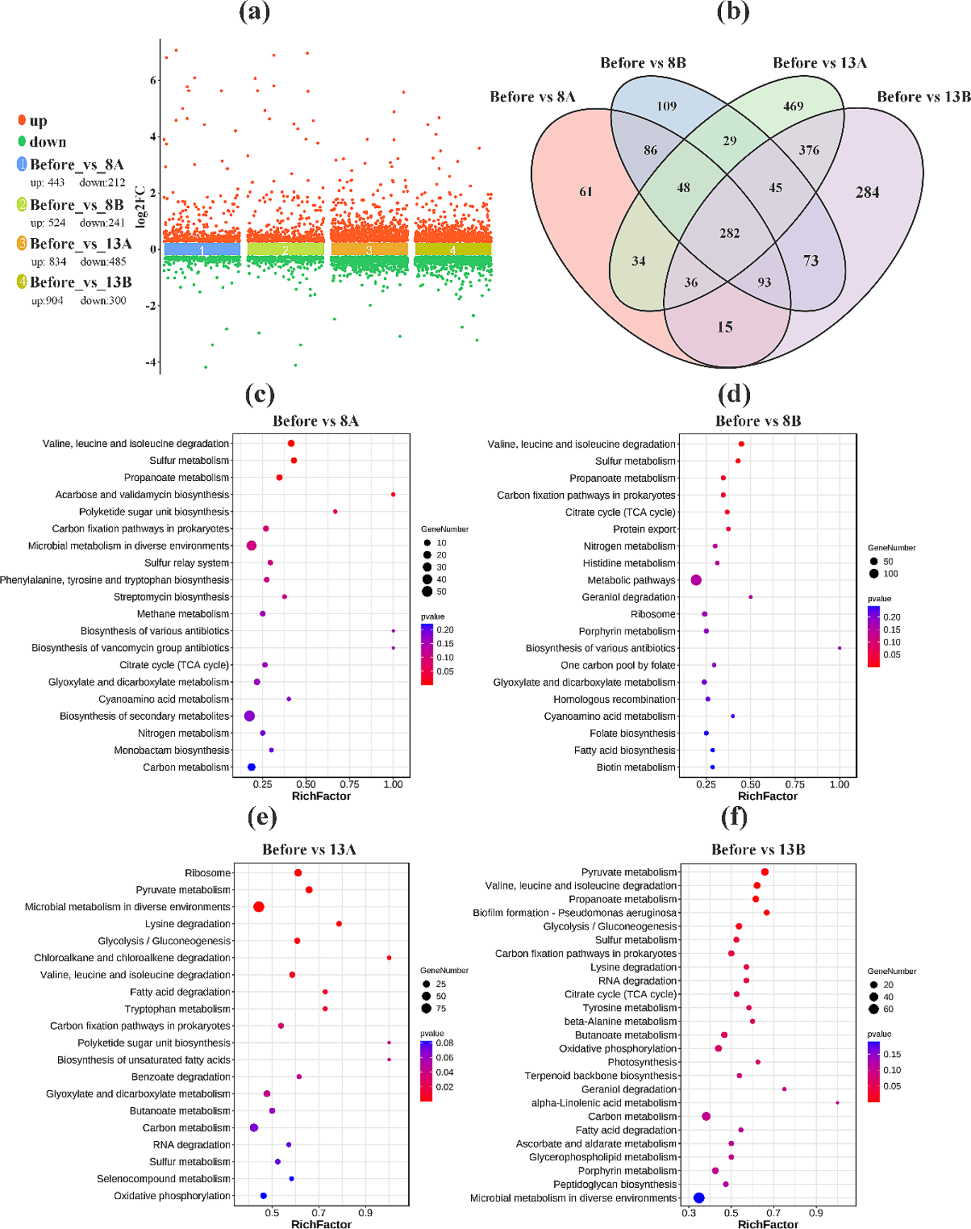
Scatter plot of DEGs **(a)**. Venn diagram of DEGs **(b)**. And KEGG pathway enrichment analysis. **(c)**, **(d)**, **(e)**, and **(f)** represent the KEGG enrichment analysis results for the Before vs 8 A, Before vs 8B, Before vs 13 A, and Before vs 13B comparison groups, respectively

In this research, four comparison groups were established: Before vs. 8A, Before vs. 8B, Before vs. 13A, and Before vs. 13B. Here, “Before” represents the 5 min pre-shock group, “8A” and “8B” represent 5 and 10 min after an 8% NaCl shock, while “13A” and “13B” represent 5 and 10 min after a 13% NaCl shock. The Before vs 8 A and Before vs 8B comparison groups had 655 (443 upregulated and 212 downregulated) and 765 (524 upregulated and 241 downregulated) DEGs, while the Before vs 13 A and Before vs 13B comparison groups had 1319 (834 upregulated and 485 downregulated) and 1204 (904 upregulated and 300 downregulated) DEGs, respectively (Fig. 4a). Furthermore, Wayne plots displayed in Fig. 4b demonstrated 509 common DEGs among Before vs 8 A and Before vs 8B comparison groups, representing 77.8% and 66.5% of their total DEGs, respectively. On the other hand, Before vs 13 A and Before vs 13B comparison groups shared 739 common DEGs, accounting for 56.0% and 61.4% of their respective total DEGs.

### GO Enrichment Analysis of DEGs

Further, the obtained DEGs were subjected to GO enrichment analysis. As shown in Figure S5, DEGs from the Before vs 8 A and Before vs 8B comparison groups were predominantly enriched in two categories: Biological Process (BP) and Molecular Function (MF). Sorted based on the number of DEGs, the top five GO terms were Cellular Process (GO:0009987), Metabolic Process (GO:0008152), Catalytic Activity (GO:0003824), Binding (GO:0005488), and Transporter activity (GO:0005215). In addition to the mentioned GO terms above, GO terms including response to chemical (GO:0042221) (*p* = 0.039) and response to toxic substance (GO:0009636) (*p* = 0.039) were found in the Before vs 13 A comparison group (Additional file 1: Table S4). Additionally, an upregulated DEG (HELO_RS18165, peroxiredoxin) was enriched in the GO term Antioxidant activity (GO:0016209) among both Before vs 8 A and Before vs 8B comparisons. However, in the comparisons of Before vs 13 A and Before vs 13B, there was no enrichment of upregulated DEGs in Antioxidant activity (GO:0016209), and 6 and 3 downregulated DEGs were observed, respectively (Additional file 1: Table S4).

### KEGG Pathway Enrichment analysis of DEGs

DEGs in Before vs 8 A and Before vs 8B comparisons were mainly enriched in the KEGG level-1 categories of “Metabolism”, “Genetic Information Processing”, “Environmental Information Processing”, and “Cellular Processes”. At KEGG level-2, the enrichment included categories such as “Global and overview maps”, “Amino acid metabolism”, “Carbohydrate metabolism”, “Energy metabolism”, and “Membrane transport” (Additional file 1: Fig. S6a, b). In addition to the above enrichments, DEGs in Before vs 13 A and Before vs 13B comparisons were also enriched in KEGG level-1 categories of “Organismal Systems” and KEGG level-2 category “Environmental adaptation” (Additional file 1: Fig. S6c, d).

**Fig. 5:**
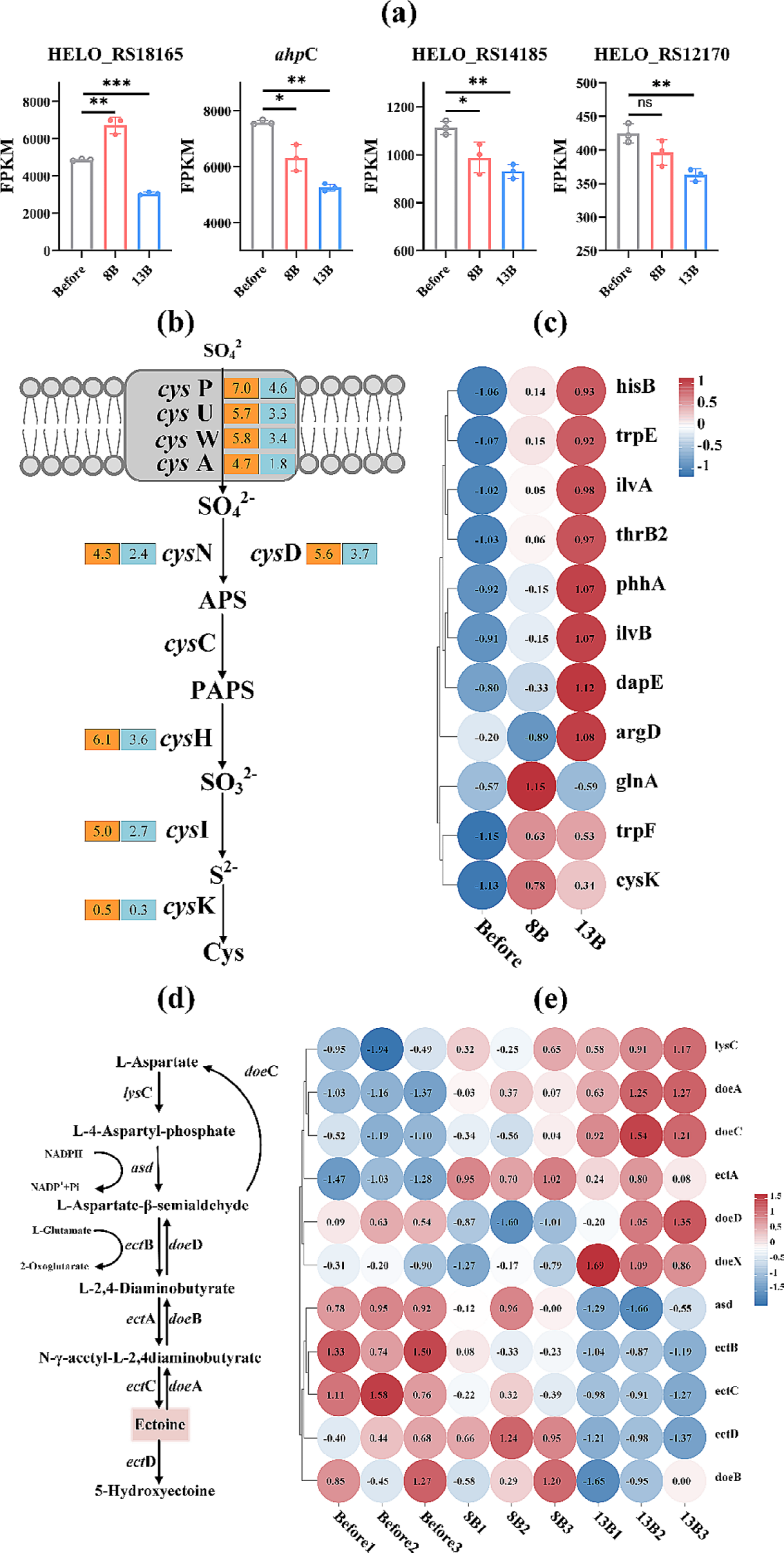
The expression level of four peroxiredoxin coding genes **(a)**. The Log_2_FC of key genes in sulfate metabolism, orange and blue rectangles with number represent gene expression changes in the Before vs Shock8B and Before vs Shock13B **(b)**. Heatmap of the expression levels of upregulated DEGs in the amino acid biosynthesis pathways during the Before vs 8B and Before vs 13B comparisons **(c)**. Schematic diagram of the ectoine metabolism **(d)**. Heatmap of the expression levels of key genes involved in the ectoine metabolism in the Before, 8B, and 13B groups **(e)**. * represents *p* < 0.05, ** represents *p* < 0.01, *** represents *p* < 0.0005

Moreover, DEGs in the Before vs 8 A comparison group were significantly enriched in “Valine, leucine, and isoleucine degradation (ko00280)”, “Sulfur metabolism (ko00920)”, and “Propanoate metabolism (ko00640)”. Two more significantly enriched pathways including “TCA cycle (ko00020)” and “Carbon fixation pathways in prokaryotes (ko00720)” were found in the Before vs 8B comparison group (Fig. 5c, d). Apart from these five mentioned enriched pathways, additional significantly enriched pathways were found in both the Before vs 13 A and Before vs 13B comparisons. These pathways include “Pyruvate metabolism (ko00620)”, “Biofilm formation - *Pseudomonas aeruginosa* (ko02025)”, and “Glycolysis/Gluconeogenesis (ko00010)”.

### Changes in antioxidant pathways after NaCl shock

The results showed a significant increase in ROS and MDA levels after NaCl shock, indicating that apart from osmotic stress NaCl shock also induced oxidative stress in the cells. The transcriptome analysis revealed that in both Before vs 8B and Before vs 13B comparisons, there were 2 upregulated and 1 downregulated DEGs were enriched in Glutathione metabolism (ko00480). *GSR* (glutathione reductase), which catalyzes the conversion of oxidized glutathione (GSSG) to reduced glutathione (GSH), was downregulated in 8B (-0.3-log_2_FC) and 13B (-0.4-log_2_FC), respectively. The remaining two upregulated DEGs, *ggt* (glutathione hydrolase) and *cha*C (glutathione-specific gamma-glutamylcyclotransferase), which can break down GSH into amino acids, exhibited upregulation with log_2_FC values of 3.0 and 0.5 in 8B, and 4.3 and 0.3 in 13B, respectively. Another notable distinction related to peroxiredoxin coding genes was identified in *H. elongata*, where four genes (HELO_RS14185, HELO_RS18165, *ahp*C, HELO_RS12170) were pinpointed. Particularly, HELO_RS18165 exhibited substantial upregulation in 8B (0.5 -log_2_FC) and high expression levels (FPKM ranging from 4800 to 7000) (Fig. 5a). HELO_RS18165 encodes the 1-*Cys* peroxiredoxin, which is crucial antioxidant enzymes in bacterial defense against oxidative damage induced by ROS. Moreover, the peroxidase (POD) and catalase (CAT) enzyme activities were also determined, revealing a significant increase in the 8B group, while the enzyme activity of glutathione-s-transferase (GST) showed no significant change after NaCl shock (Additional file 1: Fig. S3d).

Moreover, eight key genes involved in Sulfur metabolism (ko00920) were significantly upregulated in both Before vs 8B and Before vs 13B comparisons. These genes are associated with sulfate transport (*cys*P, 7.0 and 4.6-log_2_FC; *cys*U, 5.7 and 3.3-log_2_FC; *cys*W, 5.8 and 3.4-log_2_FC; *cys*A, 4.7 and 1.8-log_2_FC), sulfate activation (*cys*D, 5.6 and 3.7-log_2_FC; *cys*N, 4.5 and 2.4-log_2_FC), 3’-Phosphoadenosine 5’-phosphosulfate (PAPS) reduction (*cys*H, 6.1 and 3.6-log_2_FC), sulfite reduction (*cys*I, 5.0 and 2.7-log_2_FC), cysteine synthase (*cys*M, 0.5 and 0.3-log_2_FC), and the positive regulator of cysteine biosynthesis (*cys*B, 1.9 and 0.8-log_2_FC). The values after each gene represented the comparison of gene expression in Before vs 8B and Before vs 13B (Fig. 5b). The increased cysteine content after shock was also consistent with the enhancement of Sulfur metabolism pathway.

### Changes in pathways related to osmotic stress resistance after NaCl shock

Intracellular amino acid levels exhibited a rapid response to NaCl shock, whereas the corresponding gene expression response was less pronounced. As illustrated in Fig. 5c, there were 7 and 10 upregulated DEGs identified in the amino acid biosynthesis pathways during the Before vs 8B and Before vs 13B comparisons, respectively. These DEGs include *ilv*B, *ilv*A, *his*B, *trp*E, *trp*F, *phh*A, *thr*B, *arg*D, *dap*E, *gln*A, and *cys*M, contributing to the biosynthesis of isoleucine, histidine, tryptophan, phenylalanine, threonine, arginine, lysine, glutamine, and cysteine, respectively. Notably, *gln*A (glutamine synthetase) exhibited a noteworthy upregulation specifically in 8B (0.5-log_2_FC), aligning with the marked increase in glutamine content after 8% NaCl shock.

*H. elongata* utilizes the *Trk*H and *Trk*I transport systems to accumulate potassium ions in the cytoplasm [26]. Surprisingly, the expression of these two genes was not increased in 8B compared to the Before group (Additional file 1: Fig. S3b).

In the ectoine biosynthesis pathway, when comparing Before vs 8B and Before vs 13B, *ect*A showed an upregulation of 0.4 and 0.3-log_2_FC, respectively. However, *ect*B and *ect*C exhibited downregulation with values of -0.2 and − 0.3-log_2_FC in 8B, and − 0.3 and − 0.6-log_2_FC in 13B (Fig. 5e). In the comparison of Before vs 13B, both *doe*A and *doe*C genes in the ectoine degradation pathway exhibited a significant upregulation of 0.7-log_2_FC, alongside enhanced expression of the transcription factor *doe*X associated with the pathway (Fig. 5e). These results suggest that the ectoine biosynthesis in 8B and 13B groups was not enhanced at the transcriptional level, while the degradation pathway was enhanced in 13B group.

### Changes in central carbon metabolism and oxidative phosphorylation pathways after NaCl shock

**Fig. 6:**
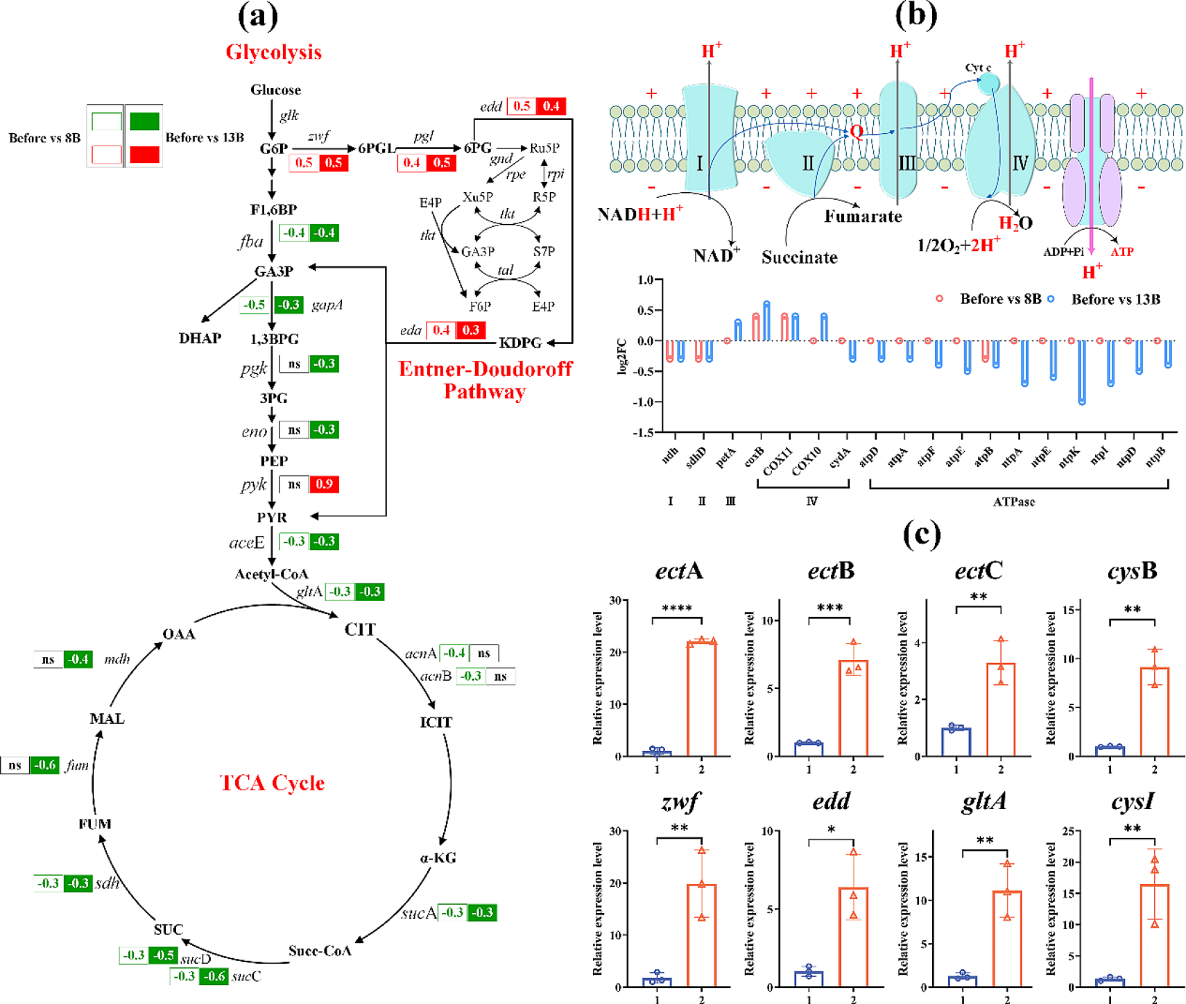
The DEGs in central carbon metabolism **(a)**. Numbers represent the log_2_FC of DEGs in Before vs 8B and Before vs 13B comparisons. The expression levels of genes in oxidative phosphorylation pathway **(b)**. qRT-PCR analysis of eight genes in *H. elongata* at one hour after 8% NaCl shock **(c)**. Where the abscissa (1 and 2) indicates different sampling time, which are 5 min before shock and 60 min after shock, respectively. * represents *p* < 0.05, ** represents *p* < 0.01, *** represents *p* < 0.0005. **** represents *p* < 0.0001

In the central carbon metabolism, the glycolysis and TCA cycle showed enrichment of 2 and 8 downregulated DEGs in the comparison of Before vs 8B, and 4 and 8 downregulated DEGs in the comparison of Before vs 13B (Fig. 6a). These DEGs included *fba* (-0.4 and − 0.4-log_2_FC), *gap*A (-0.5 and − 0.3-log_2_FC), *pgk* (ns and − 0.3 log_2_FC), and *eno* (ns and − 0.3 log_2_FC) in glycolysis pathway, *ace*E (-0.3 and − 0.3-log_2_FC), *glt*A (ns and − 0.3-log_2_FC), *acn*A (-0.3-log_2_FC and ns) *acn*B (-0.3 and ns-log_2_FC), *suc*A (-0.4 and − 0.3-log_2_FC), *suc*C (-0.3 and − 0.6-log_2_FC), *suc*D (-0.3 and − 0.5-log_2_FC), *sdh*D (-0.3 and 0.3-log_2_FC), *fum*C (ns and − 0.6-log_2_FC), and *mdh* (ns and − 0.4-log_2_FC) in TCA cycle (Fig. 6a). Furthermore, in both comparisons, the Entner-Doudoroff (ED) Pathway exhibited enrichment of 4 upregulated DEGs, namely *zwf* (0.5 and 0.5-log_2_FC), *pgl* (0.4 and 0.5-log_2_FC), *edd* (0.5 and 0.4-log_2_FC), and *eda* (0.4 and 0.3-log_2_FC) (Fig. 6a). The oxidative phosphorylation pathway exhibited 2 upregulated and 3 downregulated DEGs in 8B, and 4 upregulated and 14 downregulated DEGs in 13B (Fig. 6b). In the 8B group, the 2 upregulated DEGs were located in the cytochrome C oxidase complex IV of the respiratory chain (*COX*11 and *cox*B, both upregulated by 0.4-log_2_FC), responsible for the process of transferring electrons from cytochrome c to oxygen molecules and generating water (Fig. 6b). Additionally, the 3 downregulated DEGs were found in the ubiquinone oxidoreductase complex I (*ndh*, -0.3-log_2_FC), succinate dehydrogenase II (*sdh*D, -0.3-log_2_FC), and ATP synthase (*atp*B, -0.3-log_2_FC). In the 13B group, there were 11 downregulated DEGs in ATP synthase, and 4 upregulated DEGs in cytochrome C oxidoreductase complex III and cytochrome C oxidase complex IV (*pet*A, *COX*10, *COX*11, *cox*B) (Fig. 6b).

### Changes in other noteworthy pathways after NaCl shock

In addition, attention was given to the pathways of peptidoglycan biosynthesis and biofilm formation. The results revealed 3 and 7 upregulated DEGs in the Peptidoglycan biosynthesis (ko00550) in the comparison of Before vs 8B and Before vs 13B, respectively, including *mur*A, *mur*C, *mur*D, *mur*E, *mur*F, *mur*G, *mtg*A. The Biofilm formation - *Pseudomonas aeruginosa* (ko02025) was significantly changed in 13B group with 10 upregulated DEGs, of which 8 were associated with the Type VI secretion system (T6SS) (*imp*L, *imp*J, *imp*H, *imp*C, *imp*B, *hcp*1, *hcp*2, and *vas*G, upregulated by 0.3, 0.4, 0.5, 0.5, 0.5, 0.6, 0.7, and 1.1-log_2_FC, respectively). Furthermore, two DEGs were also found in Lipopolysaccharide synthesis pathway (ko00540) in 13B group, namely *lpx*B (lipid-A-disaccharide synthase, 0.3-log2FC) and *lpx*K (tetraacyldisaccharide 4-kinase, 0.6-log2FC). These results were consistent with the significant increase in polysaccharide content observed in the fermentation broth at 4 h following 13% NaCl shock (Additional file 1: Fig. S3c).

### Effect of exogenous added protectants on *H. elongata* cells under NaCl shock

**Fig. 7:**
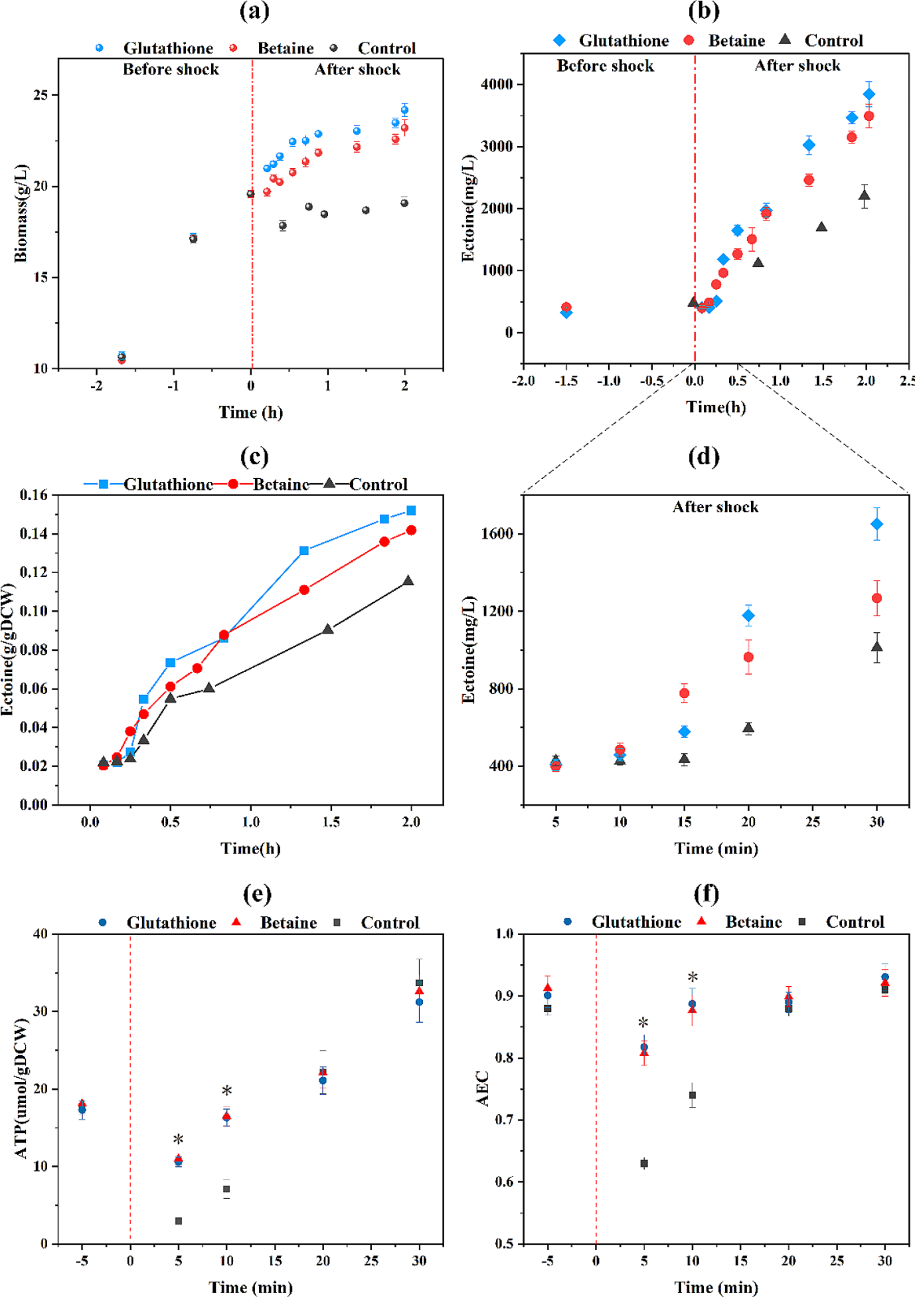
Changes in physiological parameters of *H. elongata* after 8% NaCl shock in betaine and glutathione added groups. Biomass **(a)**, ectoine production **(b)** and **(d)**, ectoine produced per biomass **(c)**. And the changes in ATP content **(e)** and AEC ratio **(f)**. * represents *p* < 0.05

To investigate the impact of exogenous added protectants on ectoine biosynthesis, experiments were conducted within the 8% NaCl shock group. Specifically, the osmoprotectant betaine and the antioxidant glutathione were added 10 min before shock treatment at a concentration of 2 g/L. They were assigned to the groups labeled C, G, and B, representing the control, glutathione-added, and betaine-added groups, respectively. The results demonstrated no substantial decrease in biomass after NaCl shock in both experimental groups (Fig. 7a), with a continual increase in biomass, reaching 19.08 g/L (C), 24.19 g/L (G), and 23.20 g/L (B) at 2 h after shock. The ectoine content in the experimental group exhibited significant elevation compared to the control group after shock, reaching 3.85 (G) and 3.49 (B) g/L at 2 h after shock (Fig. 7b). Moreover, within 15 min following the shock, the ectoine content in the experimental groups underwent a rapid increase and reached 578.55 ± 30 (G) and 777.27 ± 49 (B) mg/L, which were 1.33 and 1.79 times higher than that of the control group (434.45 ± 31 mg/L), respectively (Fig. 7d). Additionally, *p*_ectoine_ levels were significantly higher in both experimental groups than in the control group (Fig. 7c). Furthermore, despite a decrease in ATP contents and AEC ratios in the experimental groups at 5 and 10 min after salt shock, they were significantly higher than those in the control group (AEC consistently greater than 0.8) (Fig. 7e, f). Besides, the MDA content at 10 min after shock was determined in the group with glutathione supplementation. The results indicated a significant decrease in MDA content compared to the control group, with no significant difference observed compared to the before shock group (Additional file 1: Fig. S3d).

### The validation of RNA-Seq Data by qRT-PCR

In the current research, a set of eight genes associated with sulfur metabolism and ectoine biosynthesis were chosen for qRT-PCR analysis to validate the relative expression levels observed in the RNA-seq data. The validation was performed using the Pearson correlation coefficient (r). The outcomes of the correlation analysis (Additional file 1: Fig. S7) demonstrated r-values spanning from 0.81 to 0.98, underscoring the substantial concordance between the qRT-PCR outcomes and the transcriptome patterns. Furthermore, the expression levels of eight key genes at 1 h after 8% NaCl shock were also determined by qRT-PCR. Compared to before shock, the expression levels of *ect*A, *ect*B, and *ect*C genes involved in ectoine biosynthesis significantly increased by 22.0, 7.1, and 3.3 times, respectively, one hour after 8% NaCl shock (Fig. 6c). Additionally, genes related to sulfur metabolism, *cys*B, and *cys*I, showed significant upregulation by 9.1 and 12.5 times, while the gene expression levels of *zwf*, *edd*, and *glt*A were also upregulated by 11.3, 6.3, and 8.7 times, respectively (Fig. 6c).

## Discussion

Salinity fluctuations present a daily challenge for microorganisms [27]. *Halomonas elongata*, a moderately halophilic bacterium with a broad NaCl tolerance range (1-16%) [13, 19], serves as an exemplary model for studying the stress response to NaCl shock. This study investigated the stress response of *H. elongata* to NaCl shock by utilizing a multi-parameter online detection platform for continuous monitoring of physiological changes. Subsequent analysis of the transcriptome and metabolites at a short time scale unveiled the response strategies of *H. elongata* to varied shock pressures induced by NaCl concentrations (1% to 5%, 8%, and 13%).

Under 5% NaCl shock, *H. elongata* exhibited high robustness, quickly restoring its growth rate and ultimately achieving the highest biomass. This finding is consistent with the previous study indicating a maximum specific growth rate under 6% NaCl condition [19]. When exposed to 8% NaCl, *H. elongata* required a longer recovery time for the cell growth, while the ectoine content rapidly increased after shock and reached the highest level. This finding is also consistent with previous research, where the highest ectoine production was observed under 8% NaCl condition [19]. Notably, the obtained maximum ectoine productivity (1450 ± 99 mg/L/h) in 8% NaCl shock was substantially higher than that in other halophiles and even exceeded its value (1130 ± 20 mg/L/h) under normal cultivation in 8% NaCl [19]. However, treatments ranging from 1% to 13% NaCl shock exceed the maximum capacity of *H. elongata*, exerting strong inhibitory effects on cell growth and ectoine biosynthesis. Moreover, the inhibitory effects on cell respiration aggravated with the increased NaCl concentration, similar to the observed reduction in the respiratory rate of *Saccharomyces cerevisiae* under osmotic challenges [28]. Furthermore, following the NaCl shock, the glucose consumption rates decreased while the cell metabolism became more efficient, resulting in higher biomass yield and reduced CO_2_ generation. Similar high metabolic efficiency under salt stress was also observed in a previous study on the halophilic bacterium *Chromohalobacter salexigens* [29].

### The osmoregulatory mechanisms in *H. elongata* after NaCl shock

After 8% NaCl shock, intracellular ectoine content did not significantly increase until 20 min after the shock. However, amino acids exhibited a notably faster response, with 15 amino acids increasing within 5 min after shock. This finding is consistent with the crucial role of amino acids in maintaining cellular osmotic pressure under salt stress [30, 31], and the elevation in amino acid contents during the early stage is often observed as a response to salt stress [32]. Besides functioning as osmolytes in many microorganisms [33], glutamine also serves as a protective agent against oxidative stress [34, 35]. This may explain the larger intracellular pools of glutamine and the more significant increase observed. These results were validated by the upregulation of amino acid metabolism pathways, including multiple genes related to amino acid biosynthesis such as *gln*A, as indicated in the transcriptome analysis within 10 min after 8% NaCl shock. Moreover, the K^+^ ion exhibited an increasing trend for at least one hour after shock, aligning with another study on the role of K^+^ in osmoregulation of the *H. elongata*, which demonstrated that the increase in K^+^ ion persisted for a prolonged period (> 2 h) rather than being transient as described in non-halophiles [20]. Furthermore, the observed increase in Na^+^ levels indicated that Na^+^ also served as an additional solute for rapid osmotic balance in *H. elongata* following the shock. Interestingly, the Na^+^/K^+^ ratio (3.74) after shock seems to be too high compared with the non-halophilic bacteria. Considering the cytotoxic effects of high free Na^+^ concentration, it is reasonable to expect a significant portion of this sodium to be bound to cell structures [18]. This is supported by the observation that some structures (such as protein) in *H. elongata* become more acidic at higher salt concentrations, enhancing their cation binding capacity [18]. It should be noted that the determination of sodium ions did not differentiate between their free and bound forms. Nevertheless, it is speculated that the intracellular concentration of free sodium ions in *H. elongata* remains significantly higher than in other gram-negative bacteria.

Typically, the “salt-in” strategy is found in halophilic Archaea, wherein KCl is used to osmotically balance their cytoplasm [3]. This strategy incurs lower energy costs compared to the biosynthesis of organic osmotic solutes [36]. The occurrence of this strategy in *H. elongata* may be related to the energy crisis caused by NaCl shock in the short term (see discussion later). Notably, the NaCl shock could directly regulate the activity of potassium transport proteins [20], as no upregulation of potassium transport protein gene (*trk*A, *trk*H) expression was detected in the transcriptome results within 10 min. With the elevation of ectoine, the K^+^ ion and glutamine contents showed a declining trend. Moreover, the strong upregulation of *ect*A, B, and C at 1 h following 8% NaCl shock also demonstrated the enhancement of ectoine biosynthesis. In summary, the “salt-in” strategy and amino acids response were employed in *H. elongata* at the early stage following the NaCl shock to balance osmotic pressure. Over time, the predominant strategy shifts towards ectoine in osmotic regulation, yet the “salt-in” strategy remains crucial.

### The faster transcriptional response to oxidative stress after NaCl shock

Following the 8% NaCl shock, *H. elongata* experienced not only the osmotic stress but also oxidative stress, as evidenced by the significant increase in ROS and MDA levels following the shock. To counteract intracellular ROS, cells efficiently eliminate cytosolic H_2_O_2_ through highly active and abundant peroxidases, namely peroxiredoxins (PRXs) and glutathione peroxidases (GPXs) [37]. These peroxidases obtain their reducing power from the disulfide reductase systems [37]. In this study, a peroxiredoxin coding gene (HELO_RS18165, encoding 1-*Cys* peroxiredoxin) was significantly upregulated after 8% NaCl shock, while the GSR (Glutathione reductase), a key member of the glutathione antioxidant defense system [38], was downregulated. These results were consistent with the significant increase in POD enzyme activity after shock, while GST enzyme activity showed no significant change. This suggests that PRXs, rather than GPXs, play pivotal roles in *H. elongata* response to the oxidative stress induced by NaCl shock. Meanwhile, the increased enzymatic activity of CAT contributes to the antioxidative defence. Additionally, the cysteine residue, serving as the site of oxidation by peroxides [39], participates in the reduction of ROS catalyzed by PRXs. The significantly enhanced Sulfur metabolism after NaCl shock would strengthen the supply of cysteine, as confirmed by the elevated cysteine content observed after the shock. Thus, the enhanced Sulfur metabolism after NaCl shock could be considered a response to oxidative stress [40, 41], and this response may be regulated by the transcription factor *cys*B. Moreover, *cys*B acts as a positive regulator that enhances the expression of numerous *cys* regulon genes involved in thiosulfate and sulphate transportation, sulphate reduction, and ultimately controls cysteine biosynthesis in many bacteria [42]. In this study, *cys*B is significantly upregulated along with *cys*P, U, W, A, D, I, H, N, and M after NaCl shock. The strong and transient upregulation of *cys*B and genes involved in sulfur metabolism and cysteine biosynthesis was also observed in *E. coli* when exposed to a sublethal concentration of hydrogen peroxide [43]. *cys*B only showed upregulation at 10 min after treatment, with no significant difference at 60 min [43]. However, in this study, the upregulation of *cys*B persisted at least 1 h after 8% NaCl shock, indicating the long-lasting oxidative stress triggered by NaCl shock [25]. Therefore, it is speculated that the oxidative stress induced by NaCl shock enhances the expression of the transcription factor *cys*B, which subsequently regulates the synthesis of cysteine to participate in antioxidant defense.

### The energy crisis induced by NaCl shock negatively regulates the ectoine biosynthesis

The delayed response of ectoine, the most crucial osmoprotectant in *H. elongata*, prompts reflection on what causes the delayed initiation of ectoine biosynthesis after the shock. Notably, the delayed ectoine biosynthesis was not observed in the previous study [20] on *H. elongata* under NaCl shock (3%-6% NaCl), possibly due to the higher stress applied in this study (1%-8% NaCl). NaCl shock treatment poses a challenge to the maintenance of cell structure and integrity in short time, which is consistent with the observation of damaged cells with irregular morphology after shock. This phenomenon was also observed in another study that the plasma membrane ruptured after hyperosmotic shock [44]. The compromised integrity of the cell directly impacts the normal functioning of the respiratory chain on the cell membrane [45], leading to a reduction in ATP production. Moreover, the transcriptome results confirmed the downregulation of genes involved in complex I and ATP synthase of the respiratory chain after shock. The AEC ratio and ATP content also significantly decreased within 10 min after the shock. Previous research had estimated the energy cost of both the “compatible-solute” and “salt-in” strategy, with results indicating that the energy required for the former is much higher than that for the latter strategy [36]. This may provide an explanation for the observed adoption of the low-energy-consuming “salt-in” strategy by *H. elongata* during the early stage after 8% NaCl shock. In addition, under the presence of two protectants, the AEC ratios remained above 0.8 after shock, indicating a sustained normal energy status and possibly contributing to the quicker initiation of ectoine biosynthesis. The significant reduction in MDA content after shock with the addition of glutathione suggests that the oxidative stress induced by NaCl shock is effectively alleviated, allowing more energy to be available for the ectoine biosynthesis. Another study has also reported that the exogenous addition of glutathione enhances the adaptability of *Acidithiobacillus caldus* under NaCl stress [46]. However, the protective mechanism of glutathione still requires further exploration. To sum up, it can be speculated that the energy crisis induced by NaCl shock inhibits the initiation of ectoine biosynthesis. This also explains why there was no increase in ectoine after the 13% NaCl shock, namely, due to the prolonged compromised energy status (AEC<0.6) induced by the strong inhibition of the respiratory chain and ATP synthase.

### Future prospects

The study investigates the stress response of *H. elongata* to NaCl shock, changing from 1% to higher concentrations. Findings related to physiological changes, intracellular metabolites, and gene expressions contribute to key conclusions. Integrating various omics supports the relevance of considered parameters. Future prospects aim to enhance understanding of the stress response of *H. elongata* and optimize the process for efficient ectoine production.

Specifically, it remains to be explored what happens to cells following a 13% NaCl shock. Additionally, it is important to investigate the functions performed by the enhanced T6SS and elevated polysaccharides following 13% NaCl shock. Are there alternative, new extreme stress response mechanisms involved in maintaining cell survival? Furthermore, is the antioxidant process coupled with ectoine biosynthesis? Finally, the utilization of NaCl shock, coupled with precise regulation, has the potential to further enhance both the yield and productivity of ectoine.

## Conclusions

**Fig. 8:**
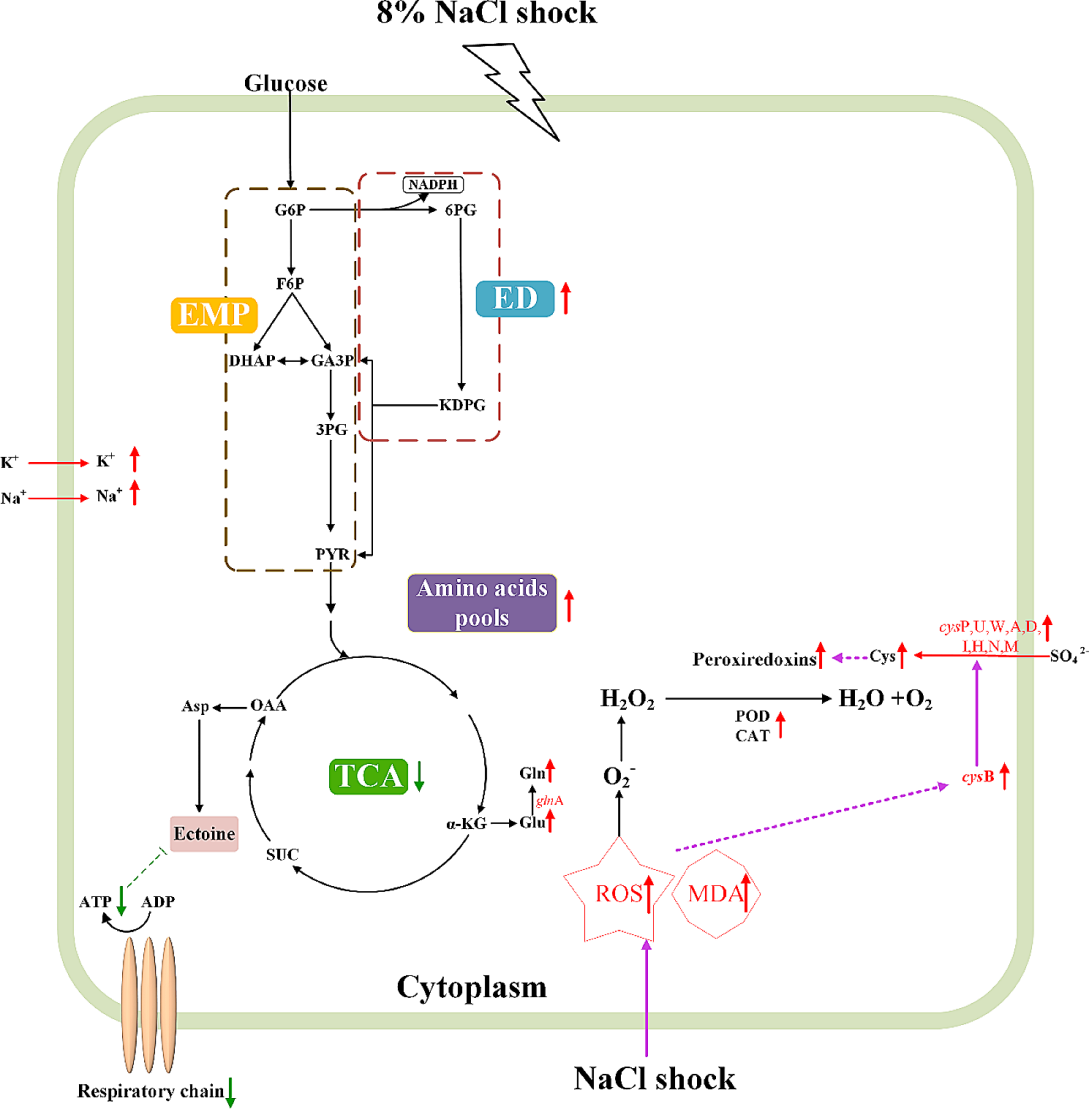
The speculated stress response mechanism to NaCl shock in *H. elongata* within short time scale after 8% NaCl shock. Red upward arrows indicate the upregulated pathways or the increased metabolite contents. Green downward arrows indicate the downregulated pathways or the decreased metabolite contents. Green T-shaped line ends denote negative effect. Purple arrows indicate a promoting effect. The solid lines indicate direct actions; the dotted lines indicate unknown mechanisms

Our study offers a comprehensive analysis of the stress response of *H. elongata* to NaCl shock at the physiological, metabolite, and transcriptome levels. Initially, *H. elongata* employs the “salt-in” strategy and augments the amino acid pool to counterbalance osmotic pressure after NaCl shock. As time progresses, the primary approach shifts towards ectoine for osmotic regulation, while the significance of “salt-in” strategy persists. In addition, NaCl shock-induced oxidative stress leads to the upregulation of sulfur metabolism and increased cysteine content, participating in the antioxidant process of PRXs. At the same time, the activities of POD and CAT enzymes are also significantly increased, constituting the antioxidant defence (Fig. 8). Furthermore, the delayed ectoine biosynthesis may be attributed to the inhibition of the respiratory chain and the consequent energy crisis caused by NaCl shock, which can be alleviated by the addition of glutathione and betaine. In conclusion, this study comprehensively examined the long-term and short-term stress responses of *H. elongata* to NaCl shock, thereby enhancing the understanding of the impact of sodium chloride on halophilic microorganisms. These findings also provide valuable theoretical insights for future advancements in optimizing industrial ectoine production.

## Materials and methods

### Bacterial strains, media, and culture conditions

The *Halomonas elongata* DSM 2581^T^, a Gram-negative bacterium, utilized in this study was generously donated by the Institute of Microbiology, Chinese Academy of Sciences.

The activation medium, seed medium and fermentation medium used in this study had been previously described in previous report [19]. For the activation process, a 30 mL plate containing the activation medium was used to activate the strain. To prepare the seed culture, a 500 mL shake flask with a working volume of 100 mL was employed. The flask was inoculated with the strain obtained from well-cultured plates, which was washed using 10 mL of sterile 8% NaCl solution. Subsequently, the seed medium was cultivated at a temperature of 37℃.

Batch fermentation was performed in a 5 L bioreactor with a working volume of 3 L. The fermentation conditions were set as follows: a temperature of 37℃, an overpressure of 0.05 MPa, and an aeration rate of 1.2 vvm. During the late-exponential growth phase, the pH was maintained at 7.0 using ammonia solution. To ensure reliable results, all experiments were conducted in triplicate.

### NaCl shock experimental procedure

During batch fermentation in 5 L bioreactor, with an initial NaCl concentration of 1% (w/v), pre-dried NaCl particles were directly added to the fermentation broth through the inoculation port in the late logarithmic phase, completing the operation within 30 s. For the 5%, 8%, and 13% NaCl shock treatments, NaCl weights of 120 g, 210 g, and 360 g were added, respectively.

### Analysis of intracellular metabolites and enzyme activity assays

Intracellular amino acids, sugar phosphates, organic acids, and sugar alcohols were quantified using gas chromatography-mass spectrometry (GC-MS) analysis (7890 A GC coupled to 5975 C MSD; Agilent Technologies, Santa Clara, CA, USA). Intracellular AMP, ADP, and ATP were quantified using liquid chromatography–mass spectrometry (LC-MS)/MS (Ultimate 3000 & TSQ Quantum Ultra, ThermoFisher Scientific Co., Ltd). The assays were consistent with the previous study [47, 48].

The content of MDA and the activities of POD, CAT, and GST enzyme were measured using commercial kits (BC0025, BC0095, BC0205, and BC0355, Solarbio), following the manufacturer’s recommendations.

### RNA extraction and transcriptome sequencing

Harvested cells from 5 min before shock (1% NaCl condition), 5 and 10 min after shock for both 8% and 13% NaCl shock conditions, during the late-exponential growth phase, were thoroughly washed three times with isotonic PBS. RNA extraction, transcriptome sequencing, and data analysis were performed at Biomarker Technologies Corporation in Beijing, China. Three biological replicates of each treatment sample, including the pre-shock condition (Before), 5 and 10 min after an 8% NaCl shock (8 A and 8B), as well as 5 and 10 min after a 13% NaCl shock (13 A and 13B), were subjected to total RNA extraction. The RNA samples were then sequenced using the Illumina HiSeq X Ten platform with a 2 × 150 bp read length, resulting in the creation of fifteen libraries.

Afterwards, gene expression abundance quantification was performed using HTSeq (version 0.9.1) and DESeq2 programs, employing default parameters. Genes meeting the criteria of an adjusted *p*-value ≤ 0.05 and |fold change| ≥ 1.2 were identified as differentially expressed. To assess the statistical enrichment of these differentially expressed genes in KEGG pathways, the KOBAS software was utilized.

### Analytical methods of cell growth, ectoine production and ROS level

Cell biomass was quantified and represented as dry cell weight (DCW). The methodology for determining DCW, along with the quantification methods for ectoine production, had been described previously [19].

Intracellular ROS levels were assessed using the oxidant-sensitive dye 2′, 7′-dichlorodihydrofluorescein diacetate (DCFH-DA, Invitrogen Detection Technologies). Following the respective treatment, cells were incubated with ten µm DCFH-DA in the dark at room temperature for 30 min and subsequently washed with PBS. Varioskan LUX multimode microplate reader (Thermo, Waltham, USA) was employed to measure the intracellular ROS levels, with an excitation wavelength of 485 nm and an emission wavelength of 530 nm.

### Quantitative real-time polymerase chain reaction (PCR) analysis

Quantitative real-time PCR (qPCR) was completed as described in(11). The primers were designed using Primer Premier 5.0 software and the listed in Table S5.

### Cell morphology and intracellular Na^+^ and K^+^ levels

Cell morphology was examined using scanning electron microscopy (SEM). After NaCl shock, collect an appropriate amount of bacterial culture, centrifuge to remove the supernatant, and wash cells thrice with isotonic PBS. Fix with 2.5% glutaraldehyde overnight at 4℃. Remove the fixative, wash with 10mM PBS three times, dehydrate with 50%, 70%, 90%, and 100% ethanol for 10 min each. Replace ethanol with 100% acetonitrile for 20 min, then freeze-dry. Attach dried samples to conductive adhesive, gold-coat, and observe using a Hitachi S3400.

Take an appropriate amount of bacterial liquid, centrifuge at 12,000 rpm for 5 min, remove the supernatant, and carefully aspirate any remaining salt solution on the tube walls. Then, scrape an appropriate amount of bacterial pellet, place it in a freeze-dryer overnight to remove moisture through freeze-drying. After drying, weigh an appropriate amount of bacterial cells, add 10 times the volume of nitric acid to the digestion tube, digest at 100℃ for 4 h, and then collect the supernatant. Intracellular levels of Na^+^ and K^+^ in the supernatant were determined using an inductively coupled plasma atomic emission spectrometer (ICP-AES).

### Electronic supplementary material

Below is the link to the electronic supplementary material.

Supplementary Material 1

## Data Availability

Data is provided within the manuscript or supplementary information files.

## Abbreviations

G6P: Glucose-6-phosphate
OAA: Oxaloacetate
F6P: Fructose-6-phosphate
6PG: 6-phosphogluconate
DHAP: Dihydroxyacetone phosphate
GA3P: Glyceraldehyde-3-phosphate
3PG: 3-phosphate-glycerate
PYR: Pyruvate
KDPG: 2-keto-3-deoxy-6-phosphogluconate
α-KG: α-ketoglutarate
SUC: Succinate
CIT: Citrate
ICIT: Isocitrate
MAL: Malate
FUM: Fumarate
S7P: Sedoheptulose 7-phosphate
E4P: Erythrose-4-phosphate
R5P: Ribose-5-phosphate
