# Supplementary material for: Temporal dynamics of stress response in Halomonas elongata to NaCl shock: physiological, metabolomic, and transcriptomic insights
Source: Microb Cell Fact. 2024 Mar 23;23:88. doi: 10.1186/s12934-024-02358-5 (PMC10960403; doi:10.1186/s12934-024-02358-5)
Supplement: Supplementary file 1 — Supplementary Material 1 [file 12934_2024_2358_MOESM1_ESM.docx]

**Additional file**

**Table S1:** **Carbon distribution within 4 hours after NaCl shock**

| Group | Control | 5% NaCl shock | 8% NaCl shock |
| --- | --- | --- | --- |
| C in Biomass (mol) | 0.35 | 1.09 | 0.38 |
| C in CO_2_ (mol) | 1.44 | 1.65 | 0.92 |
| C in consumed Glucose (mol) | 2.16 | 1.98 | 1.29 |
| △C^a^ (mol) | 0.37 | -0.77 | 0.00 |

△C^a^ represents the difference between the total carbon in consumed glucose and the carbon in the generated biomass and CO_2_.

**Table S2: The changes in purine nucleotide and energy charge during 30 min after NaCl shock**

|  | AMP  (μmol/ gDCW) | ADP  (μmol/ gDCW) | ATP  (μmol/ gDCW) | AXP^a^  (μmol/ gDCW) | AEC^b^ |
| --- | --- | --- | --- | --- | --- |
| Before^1^ | 1.5 ± 0.1 | 2.1 ± 0.2 | 17.7 ± 1.1 | 21.3 ± 1.4 | 0.88 |
| 5 min^1^ | 1.1 ± 0.1 | 1.7 ± 0.2 | 3.0 ± 0.5 | 6.1 ± 0.8 | 0.63 |
| 10 min^1^ | 1.7 ± 0.2 | 2.6 ± 0.3 | 7.1 ± 1.2 | 11.3 ± 1.7 | 0.74 |
| 20 min^1^ | 1.7 ± 0.2 | 2.9 ± 0.3 | 22.2 ± 2.8 | 26.8 ± 3.3 | 0.88 |
| 30 min^1^ | 1.8 ± 0.2 | 3.1 ± 0.3 | 33.7 ± 3.1 | 38.6 ± 3.6 | 0.91 |
| Before^2^ | 1.6 ± 0.1 | 2.4 ± 0.2 | 16.8 ± 1.6 | 20.8 ± 1.9 | 0.87 |
| 5 min^2^ | 1.5 ± 0.2 | 2.4 ± 0.2 | 2.6 ± 0.3 | 6.5 ± 0.7 | 0.58 |
| 10 min^2^ | 0.9 ± 0.1 | 1.5 ± 0.2 | 1.6 ± 0.2 | 4.0 ± 0.5 | 0.59 |
| 20 min^2^ | 0.9 ± 0.2 | 1.8 ± 0.2 | 1.6 ± 0.1 | 4.3 ± 0.6 | 0.58 |
| 30 min^2^ | 1.0 ± 0.2 | 1.4 ± 0.3 | 1.6 ± 0.1 | 4.0 ± 0.7 | 0.58 |

AXP^a^ indicates the sum of adenosine phosphates.

AEC^b^ indicates the adenylate energy charge.

Superscript “1” in the first column represents the 8% NaCl shock condition.

Superscript “2” in the first column represents the 13% NaCl shock condition.

**Table S3: Summary of the sequencing reads and read mapping**

| **Sample** | **Raw reads** | **Clean reads** | **Q20**  **(%)** | **Q30**  **(%)** | **GC (%)** | **N**  **(%)** | **Error (%)** | **Uniquely mapped (%)** | **Total**  **mapped (%)** |
| --- | --- | --- | --- | --- | --- | --- | --- | --- | --- |
| Before1 | 24684110 | 24388466 | 97.34 | 94.08 | 61.09 | 0 | 0.0256 | 98.03 | 99.39 |
| Before2 | 26786218 | 26456552 | 97.39 | 94.08 | 60.81 | 0 | 0.0256 | 97.93 | 99.33 |
| Before3 | 26346916 | 26058650 | 97.5 | 94.19 | 61.25 | 0 | 0.0254 | 97.76 | 99.35 |
| Shock8A1 | 25123278 | 24837834 | 97.37 | 94.07 | 60.85 | 0 | 0.0256 | 95.42 | 99.08 |
| Shock8A2 | 25233896 | 24969946 | 97.53 | 94.22 | 60.92 | 0 | 0.0253 | 97.01 | 99.33 |
| Shock8A3 | 26883864 | 26611364 | 97.55 | 94.3 | 60.97 | 0 | 0.0253 | 96.7 | 99.38 |
| Shock8B1 | 26082326 | 25803022 | 97.25 | 93.99 | 60.96 | 0 | 0.0257 | 94.86 | 99.34 |
| Shock8B2 | 23519242 | 23269258 | 97.62 | 94.24 | 61.01 | 0 | 0.0253 | 97.07 | 99.42 |
| Shock8B3 | 25371724 | 25108416 | 97.36 | 94.02 | 61.1 | 0 | 0.0256 | 96.93 | 99.38 |
| Shock13A1 | 63107744 | 62316512 | 97.4 | 93.84 | 60.08 | 0 | 0.0257 | 96.48 | 99.21 |
| Shock13A2 | 59218088 | 58364160 | 97.14 | 93.6 | 59.99 | 0 | 0.0261 | 94.24 | 99.3 |
| Shock13A3 | 24076462 | 23788606 | 97.21 | 93.87 | 61.02 | 0 | 0.0259 | 94.98 | 99.45 |
| Shock13B1 | 22646086 | 22346172 | 97.52 | 94.14 | 60.99 | 0 | 0.0254 | 94.7 | 99.46 |
| Shock13B2 | 23293636 | 23053984 | 97.47 | 94.07 | 60.71 | 0 | 0.0255 | 96.13 | 99.42 |
| Shock13B3 | 27041454 | 26729306 | 97.32 | 94.02 | 61.16 | 0 | 0.0257 | 96.63 | 99.45 |

**clean reads:** the number of reads after remove low-quality reads, adaptor sequences, poly A and known non-coding RNAs. **Unique mapped (%):** uniquely compares the number of reads on the reference genome and the proportion of valid reads. **Multiple mapped (%):** reads the number of reads on the reference genome in multiple comparisons and the proportion of valid reads. **Total mapped (%):** the total number of reads that can be mapped to the genome and the proportion of valid reads. Q30 and Q20 percentages are proportions of nucleotides with quality value larger than 30 and 20, respectively. GC percentage is proportion of guanidine and cytosine nucleotides among total nucleotides. N (%) demonstrates the percentage of unidentified bases.

**Table S4: GO significant enrichment analysis of differentially expressed genes (p＜0.05)**

| DEGs | GO category | GO ID | Description | Up  DEGs | Down  DEGs | P  value |
| --- | --- | --- | --- | --- | --- | --- |
| Before vs 8B | BP | 0009987 | cellular process | 117 | 52 | 0.032 |
| Before vs 13A | MF | 0005198 | structural molecule activity | 34 | 0 | 0.001 |
|  | BP | 0009987 | cellular process | 237 | 165 | 0.035 |
|  | BP | 0042221 | response to chemical | 3 | 0 | 0.039 |
|  | BP | 0009636 | response to toxic substance | 3 | 0 | 0.039 |
| Before vs 13B | MF | 0005488 | binding | 272 | 147 | 0.017 |
|  | BP | 0009987 | cellular process | 290 | 102 | 0.020 |
|  | BP | 0065007 | biological regulation | 60 | 14 | 0.022 |

BP represents Biological Process, and MF represents Molecular Function.

**Table S5: Primers for qRT-PCR**

| Primer name | Primer sequence |
| --- | --- |
| 16s rRNA-F1 | GGAAGGCGATGAGGCAAG |
| 16s rRNA-R1 | GCAGCGTTTCGACATGGTT |
| *zwf*-F2 | CCCGCTTCACCTGGATTT |
| *zwf*-R2 | AGATACTGCTGTCCCTTCATTACTT |
| *edd*-F1 | AGGCGGACATCAACCACTT |
| *edd*-R1 | TTCATCCAGGCTCTGCTCC |
| *glt*A-F2 | AACTTCTTCAAGGGTTTCCGT |
| *glt*A-R2 | GTCCAGATGGTCGTGGTAGAA |
| *ect*A-F1 | GTCAAGTCCTGTCCGCCTCT |
| *ect*A-R1 | TCGCCCTCTTCGTTGGTC |
| *ect*B-F1 | CTGACCGACGAGGAAGGG |
| *ect*B-R1 | TTGAGGTGCGGGTTGTTG |
| *ect*C-F1 | TTCCACATCACCCGCATC |
| *ect*C-R1 | GGTTTCCACTTCGCCCTC |
| *cys*H-F1 | AGTGCGGTGAACCAGACATC |
| *cys*H-R1 | ATGGATCATGGCTACAACACC |
| *cys*B-F1 | AGATTGCCCAGATGGTCAGTG |
| *cys*B-R1 | CTTCCAGGGTCAGGGGTTC |
| *cys*I-F2 | GGTCCGCTGGAGCTCAAC |
| *cys*I-R2 | ATCACATCCGTCACATCTTCG |
| *metQ*-F1 | GCAAGTGGCGGCAGACAT |
| *metQ*-R1 | TGATAGGCGTTGGCGTCC |
| *cys*P-F2 | AACACTGGGAGGCGAACC |
| *cys*P-R2 | TGGAGTAGTAGGGCGAGGC |

**Fig. S1**


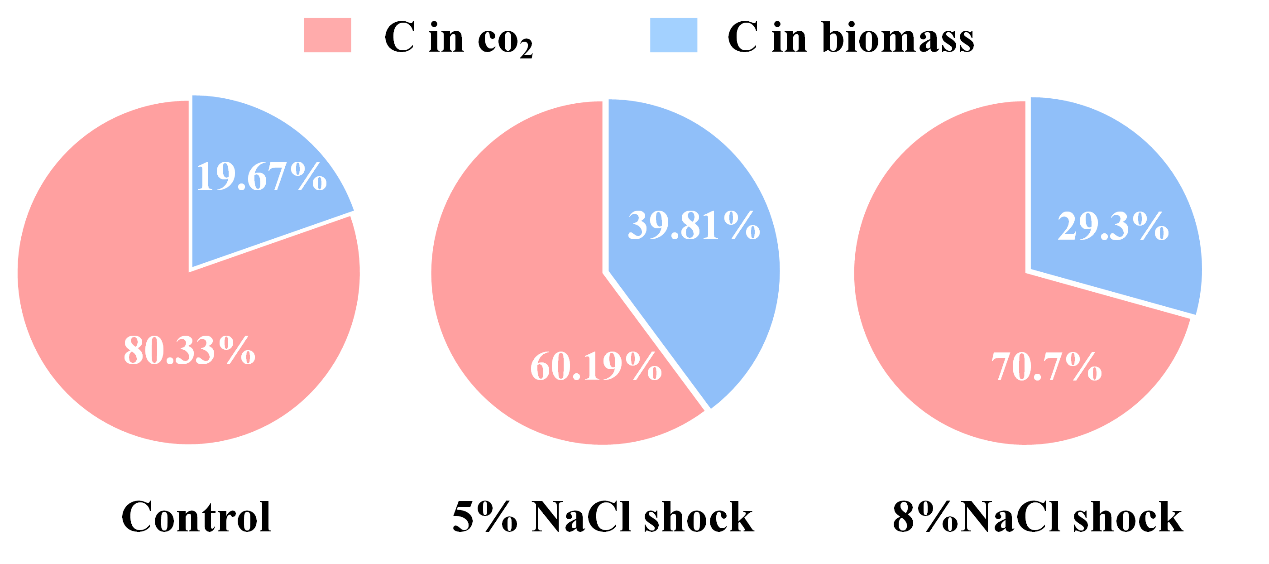


**Figure S1:** Carbon distribution within 4 hours after NaCl shock.

**Fig. S2**


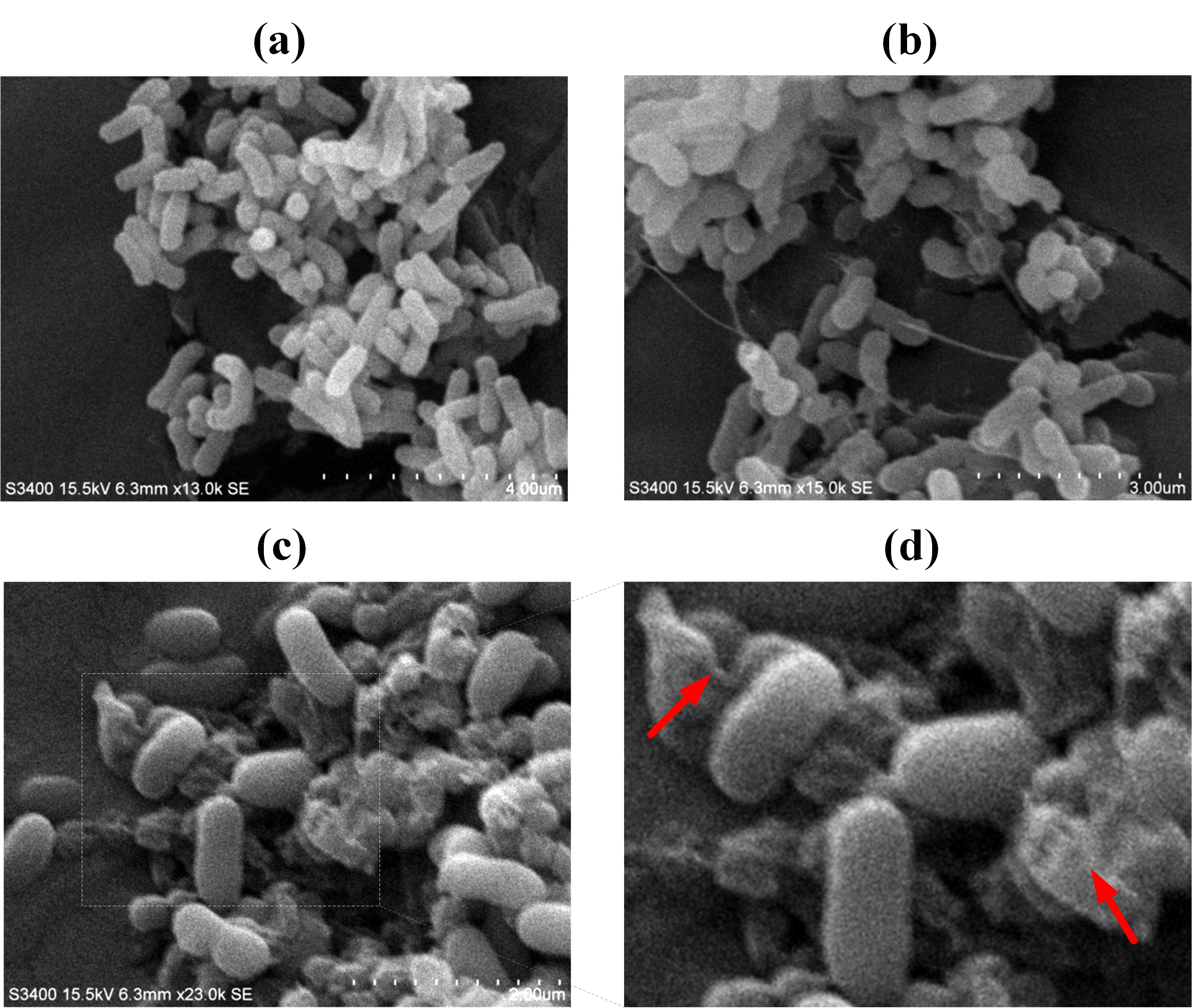


**Figure** **S2**: Scanning electron microscopy images of cells after NaCl shock. **(a)** represents cells before the shock; **(b)** and **(c)** show cells at 10 minutes after 8% and 13% NaCl shock, respectively; **(d)** represents the local enlarged drawing **(c)**, red arrows indicate some damaged cells and cell debris.

**Fig. S3**


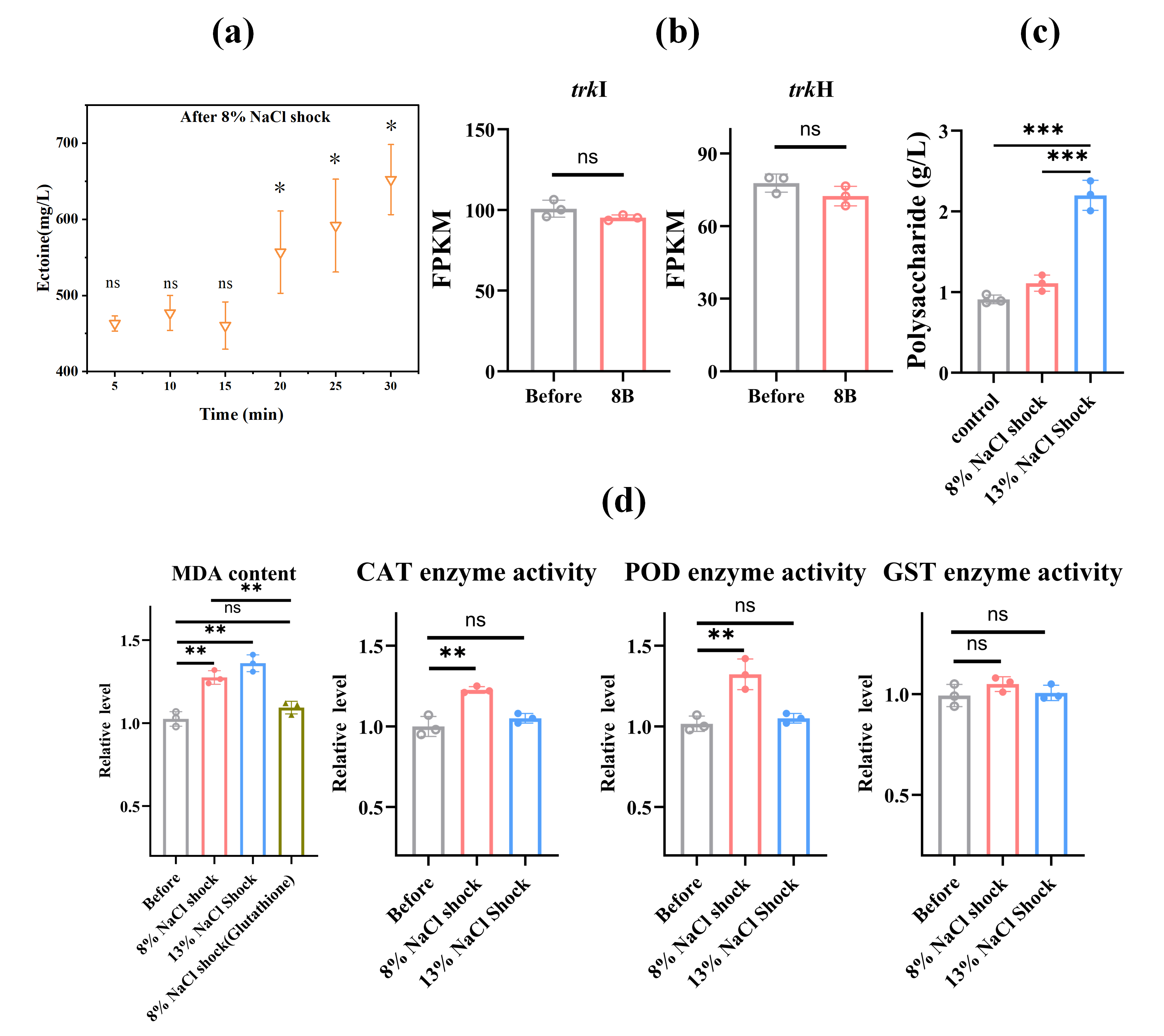


**Figure** **S3**: The ectoine content within 30 min after 8% NaCl shock **(a)**. The expression level of two genes in potassium ion transport systems **(b)**. The changes in polysaccharide content at 4 h after shock **(c)**. The changes in MDA content, POD, and CAT enzyme activities at 10 min after NaCl shock **(d)**. ** represents *p* < 0.01, *** represents *p* < 0.0005.

**Fig. S4**


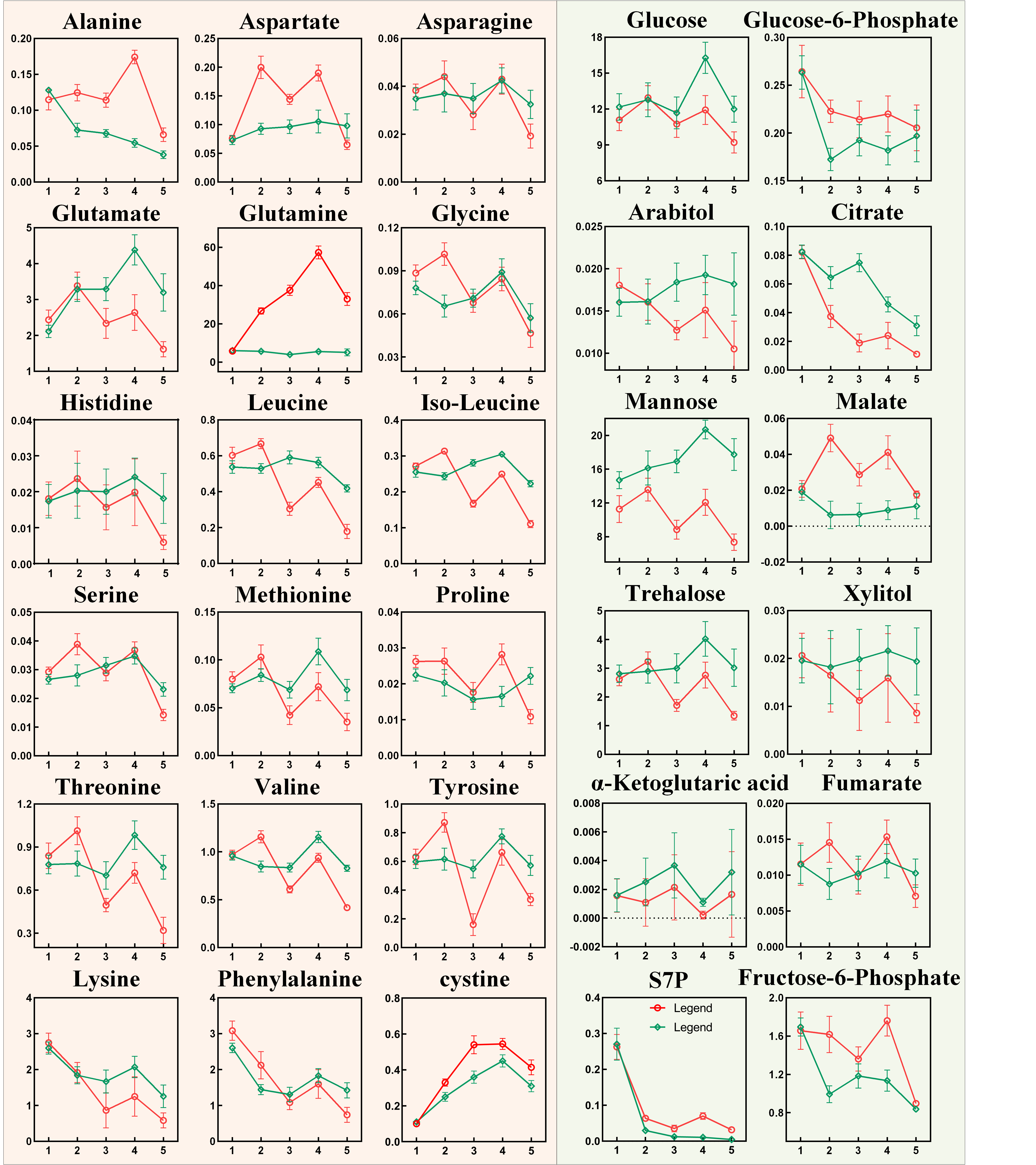


**Figure** **S4**: Changes in intracellular amino acid, sugar phosphates, organic acids, and sugar alcohols metabolites within 30 min after 8% and 13% NaCl shock. The red line represents the 8% shock group, while the green line represents the 13% shock group. The abscissa of each graph (1, 2, 3, 4, and 5) below the indicates different sampling time, which are 5 min before shock, 5, 10, 20, and 30 min after shock, respectively. The unit of vertical coordinate is mg/ gDCW.

**Fig. S5**


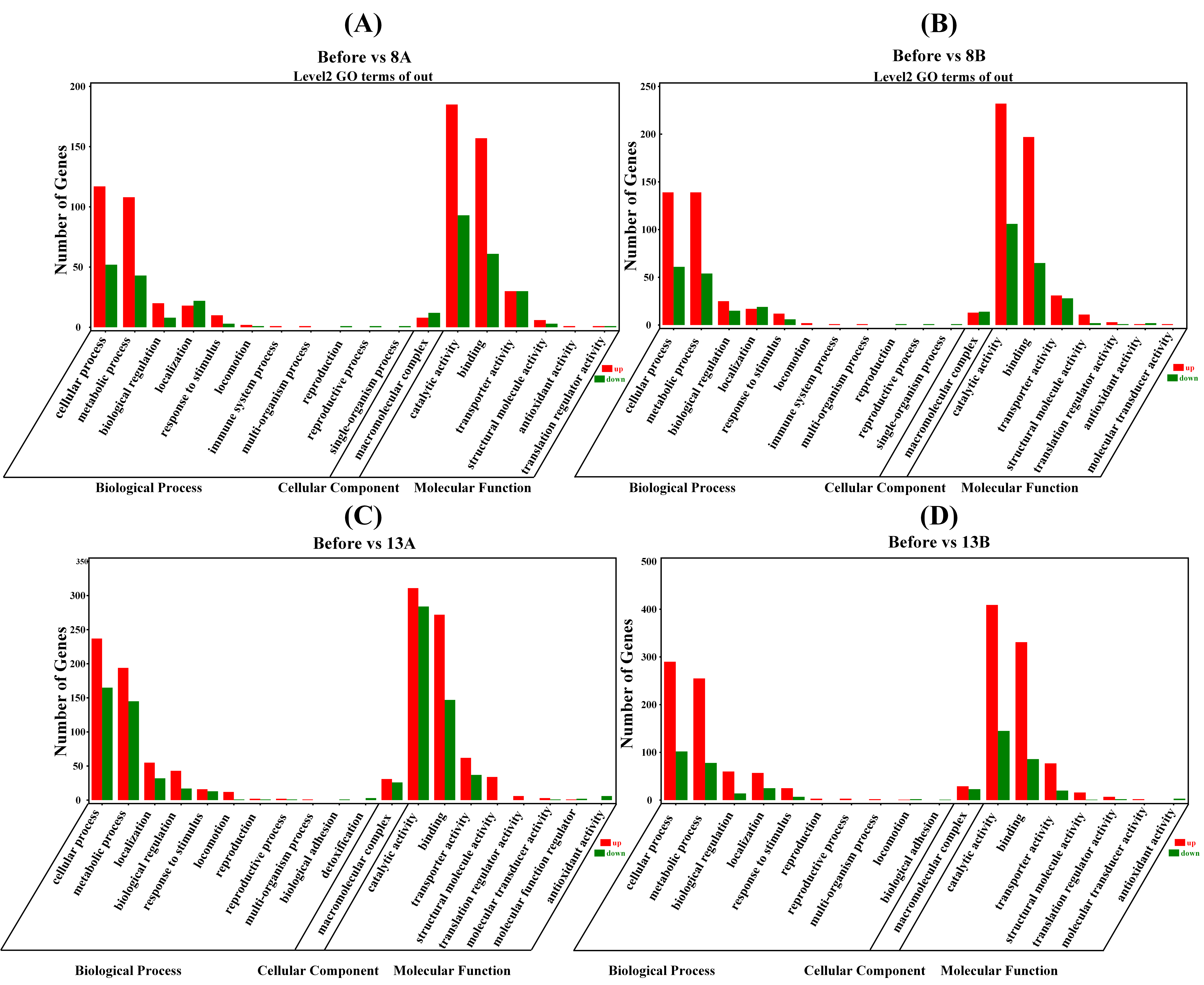


**Figure S5:** GO enrichment analysis of DEGs in four comparisons, including Before vs 8A **(a)**, Before vs 8B **(b)**, Before vs 13A **(c)**, and Before vs 13B **(d)**.

**Fig. S6**


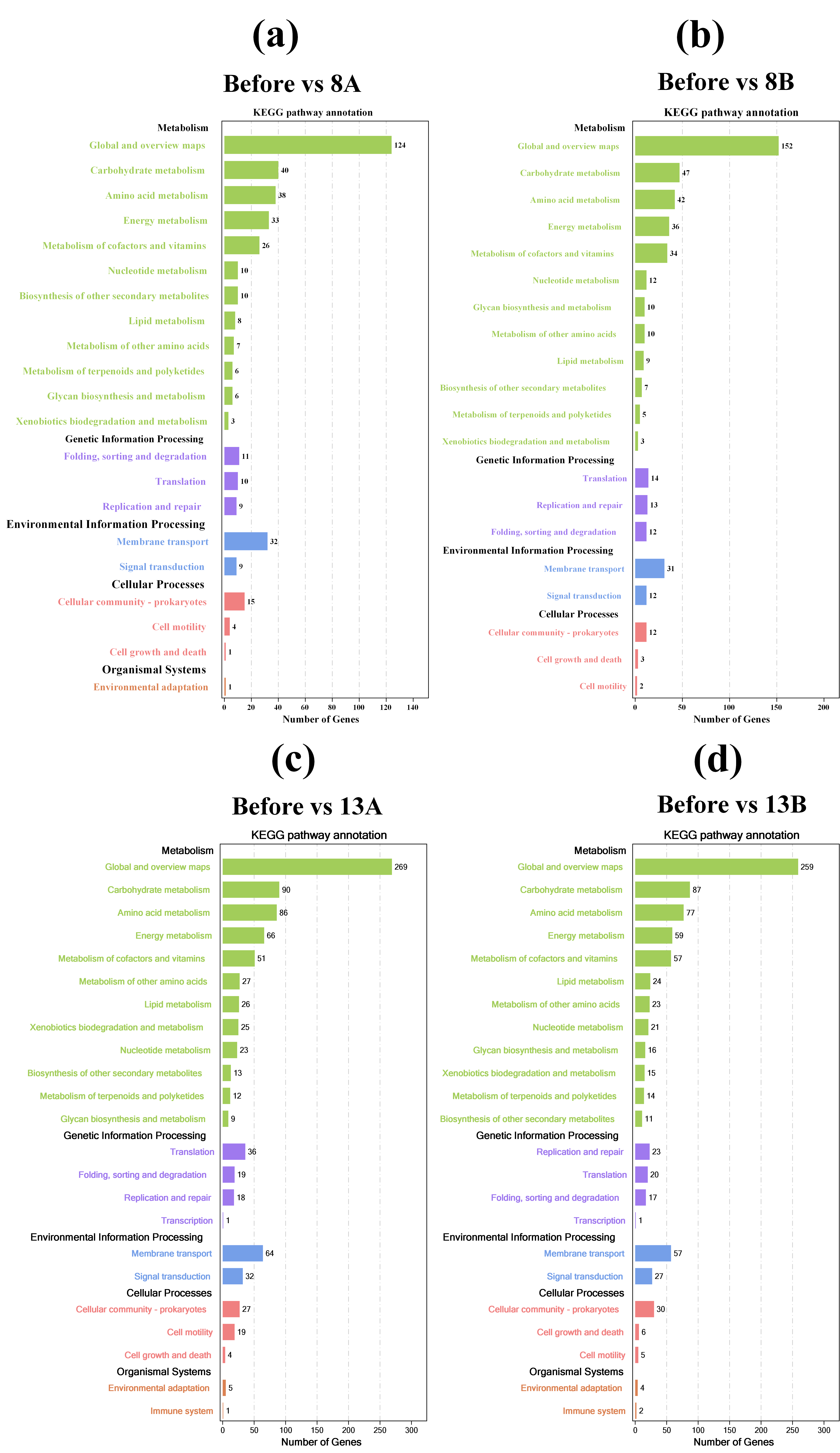


**Figure S6:** KEGG pathway enrichment analysis Of DEGs in four comparisons, including Before vs 8A **(a)**, Before vs 8B **(b)**, Before vs 13A **(c)**, and Before vs 13B **(d)**.

**Fig. S7**


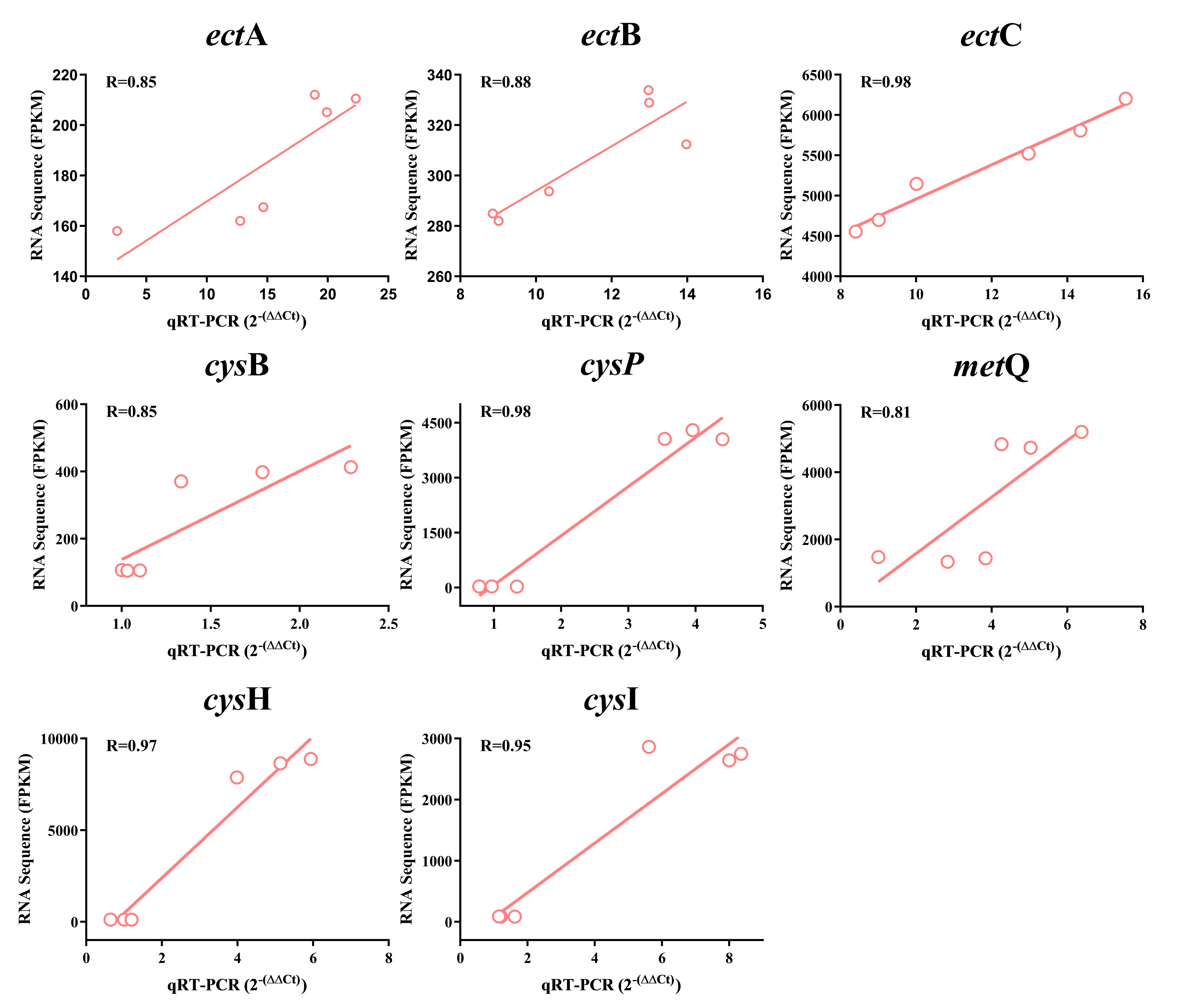


**Figure** **S7**: The expression level of eight selected genes in *H. elongata* after NaCl shock by qRT-PCR. “r”: Pearson correlation coefficient.
